# Pathogenesis of cardiomyopathy caused by variants in *ALPK3*, an essential pseudokinase in the cardiomyocyte nucleus and sarcomere

**DOI:** 10.1161/CIRCULATIONAHA.122.059688

**Published:** 2022-11-02

**Authors:** Radhika Agarwal, Hiroko Wakimoto, Joao A. Paulo, Qi Zhang, Daniel Reichart, Christopher Toepfer, Arun Sharma, Angela C. Tai, Mingyue Lun, Joshua Gorham, Steven R. DePalma, Steven P. Gygi, J.G. Seidman, Christine E. Seidman

**Affiliations:** 1 Department of Genetics, Harvard Medical School, Boston, MA 02115, USA; 2 Department of Cell Biology, Harvard Medical School, Boston, MA 02115, USA; 3 Radcliffe Department of Medicine, University of Oxford, OX3 9DU, UK; 4 Wellcome Centre for Human Genetics, University of Oxford, OX3 7BN, UK; 5 Board of Governors Regenerative Medicine Institute, Cedars-Sinai Medical Center, Los Angeles, CA, 90048 USA; 6 Smidt Heart Institute, Cedars-Sinai Medical Center, Los Angeles, CA, 90048 USA; 7 Department of Biomedical Sciences, Cedars-Sinai Medical Center, Los Angeles, CA, 90048 USA; 8 Division of Cardiovascular Medicine, Brigham and Women’s Hospital, Boston, MA 02115, USA; 9 Howard Hughes Medical Institute, Chevy Chase, MD 20815, USA

**Keywords:** cardiomyopathy, sarcomere, M-band, phosphoproteomics, pseudokinase, α-kinase, hiPSC-CM

## Abstract

**Background:**

*ALPK3* encodes α-kinase 3, a muscle-specific protein of unknown function. *ALPK3* loss-of-function variants cause cardiomyopathy with distinctive clinical manifestations in both children and adults, but the molecular functions of ALPK3 remain poorly understood.

**Methods:**

We explored the putative kinase activity of ALPK3 and the consequences of damaging variants using isogenic human induced pluripotent stem cell derived cardiomyocytes (hiPSC-CMs), mice, and human patient tissues.

**Results:**

Multiple sequence alignment of all human α-kinase domains and their orthologs revealed four conserved residues that were variant only in ALPK3, demonstrating evolutionary divergence of the ALPK3 α-kinase domain sequence. Phosphoproteomic evaluation of both ALPK3 kinase domain inhibition and overexpression failed to detect significant changes in catalytic activity, establishing ALPK3 as a pseudokinase. Investigations into alternative functions revealed that ALPK3 co-localized with myomesin proteins (MYOM1, MYOM2) at both the nuclear envelope and the sarcomere M-band. *ALPK3* loss-of-function variants caused myomesin proteins to mislocalize and also dysregulated several additional M-band proteins involved in sarcomere protein turnover, which ultimately impaired cardiomyocyte structure and function.

**Conclusions:**

ALPK3 is an essential cardiac pseudokinase which inserts in the nuclear envelope and the sarcomere M-band. Loss of ALPK3 causes mislocalization of myomesins, critical force-buffering proteins in cardiomyocytes, and also dysregulates M-band proteins necessary for sarcomere protein turnover. We conclude that *ALPK3*-cardiomyopathy induces ventricular dilatation due to insufficient myomesin-mediated force buffering and hypertrophy by impairment of sarcomere proteostasis.

## Non-standard Abbreviations and Acronyms

ALPK3: α-kinase 3
C-lobe: C-terminal lobe
CAPN3: calpain 3
DE: differentially expressed
dIC: dual interlobe cleft variant
DP: differentially phosphorylated
hiPSC: human induced pluripotent stem cell
hiPSC-CMs: human induced pluripotent stem cell-derived cardiomyocytes
IC: interlobe cleft
IVSd: interventricular septal thickness at end-diastole
LVIDd: left ventricular internal dimension at end diastole
LVPWd: left ventricular posterior wall thickness at end diastole
MuRF1/TRIM63: muscle RING-finger protein 1
MYBPC3: myosin binding protein c
MYH6: α-myosin heavy chain
MYH7: β-myosin heavy chain
MYOM1: myomesin-1
MYOM2: myomesin-2 (M-protein)
N-lobe: N-terminal lobe
sIC: single interlobe cleft variant
TMT: tandem mass tag
TRPM7: transient receptor potential cation channel 7
TTN: titin

## Introduction

Cardiomyopathies are prevalent and serious primary diseases of the heart muscle that cause premature morbidity and mortality from progressive heart failure or sudden cardiac death. Approximately half of cardiomyopathies have a genetic etiology with over 50 identified disease genes. In adults, many of these genes encode sarcomere and cytoskeletal proteins, while in children, cardiomyopathy genes often encode neuromuscular, metabolic, and mitochondrial proteins.^1^ Pathogenic variants in cardiomyopathy genes trigger one of two dichotomous cardiac morphologies: hypertrophy (increased ventricular wall thickness and mass with reduced chamber volumes) or dilatation (normal ventricular wall thickness and increased chamber volumes).^2^

One recently described cardiomyopathy arises from variants in *ALPK3*, which encodes the muscle-specific protein α-kinase 3.^3^ Recessive *ALPK3* variants (loss-of-function or deleterious missense) cause cardiomyopathy late in gestation or infancy with biventricular dilatation and depressed contractile function.^3–8^ Disease progression is often rapid with hemodynamic decompensation and death in childhood. Rare surviving children demonstrate a most atypical sequence of cardiac remodeling: biventricular dilation that transitions to biventricular hypertrophy, while contractile function remains profoundly depressed.^6^

Heterozygous *ALPK3* loss-of-function variants are also estimated to cause ~1.5-2.5% of all ventricular hypertrophy that occurs in adults.^6,9^ These heterozygous variants likely have variable and age-dependent penetrance, as obligate carrier relatives of critically ill infants with recessive *ALPK3* cardiomyopathy often have no cardiac disease.^6^

**Figure 1:**
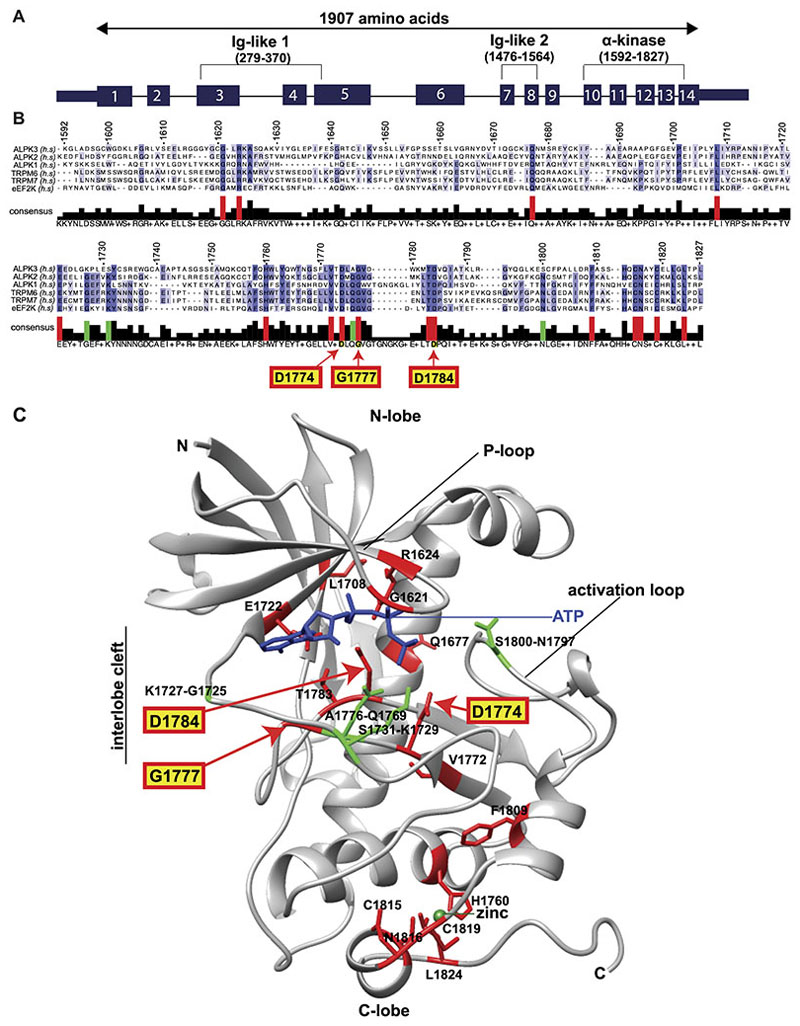
Sequence alignment of all α-kinase domains shows four nonconserved interlobe cleft residues in ALPK3. **(A)** The *ALPK3* gene contains 14 exons that encode 3 protein domains and 1907 amino acids (positions indicated in parentheses) **(B)** Sequence alignment of six human α-kinase domains (numbered by amino acid position in ALPK3) identifies 16 residues (red) that are uniformly conserved. Four residues (green) are conserved in all α-kinases (including twenty-six α-kinase orthologs) except ALPK3 (denoted ALPK3-specific nonconserved residues, see also Figure S1). The positions of three conserved residues edited by CRISPR/Cas9 (G1777E, D1784A, D1774A) are indicated with red arrows. **(C)** The positions of conserved and ALPK3-specific nonconserved residues are indicated with colors and symbols as in (B) by homology mapping to the TRPM7 α-kinase domain.^10,19^ Hyphenated residues denote amino acids in ALPK3 and TRPM7 respectively. Three of the four ALPK3-specific nonconserved residues are predicted to position ATP and bind substrate (see also Table S1). N-lobe (N-terminal lobe), C-lobe (C-terminal lobe), ATP (blue), zinc (green).

*ALPK3* encodes a 201kDa protein which contains a nuclear localization signal, two immunoglobulin-like domains, and a C-terminal α-kinase domain (Figure 1A). The α-kinase family, a subfamily of the atypical protein kinases, has six members in humans: the transient receptor potential cation channels 6 and 7 (TRPM6, TRPM7), eukaryotic elongation factor 2 kinase (eEF2K) and α-kinases 1, 2, and 3 (ALPK1, ALPK2, ALPK3).^10,11^

*ALPK3* was initially discovered during *in vitro* screens for genes that were expressed in advance of key early cardiac transcription factors (NKX2-5, GATA-4).^12^ *In situ* hybridization studies in developing mice confirmed that *ALPK3* is expressed in the early embryonic cardiac crescent and remains highly expressed in cardiomyocytes throughout life.^12^ *Alpk3*-null (*Alpk3*^-/-^) mice, generated by gene trapping, are born at normal Mendelian ratios and are viable and fertile despite biventricular hypertrophy and dilation with reduced contractility.^13^

Despite its primordial and persistent cardiac expression and clear role in cardiomyopathy pathogenesis, the molecular functions and putative kinase activity of ALPK3 remain unknown. To investigate these properties, we introduced *ALPK3* variants in isogenic human induced pluripotent stem cells (hiPSCs) that either produced targeted missense residues in the α-kinase domain or reduced ALPK3 expression. The molecular and functional effects of these variants were first characterized in hiPSC-derived cardiomyocytes (hiPSC-CMs) and then validated in a new *Alpk3* mutant mouse model and in human *ALPK3* patient tissues. Our studies reveal that the kinase domain of ALPK3 lacks catalytic activity but is important for regulating the activities of key force-buffering myomesin proteins in both the nucleus and sarcomere.

## Methods

The authors declare that all supporting data are available within the article [and its online supplementary files].

### hiPSCs

PGP1 eGFP-TTN is a male human pluripotent stem cell line with an endogenous GFP tag engineered on the N-terminus of TTN which inserts at sarcomere Z-discs.^14^ Tissue culture dishes (6-well) (Fisher Scientific, 07-200-83) were coated with BD Matrigel (BD Biosciences 354277) at a 1:320 dilution in DMEM F12 (Invitrogen 11330057). hiPSCs were seeded on Matrigel-coated tissue-culture dishes at approximately 30% confluency with daily media replacement (mTESR1, STEMCELL Technologies). hiPSCs were passaged every 3-4 days, using 0.5mM EDTA in PBS, so as to not exceed a confluency of 80%. Cell viability after passaging was improved by including 10μM ROCK inhibitor Y-27632 (R&D Systems, 125410) in culture media for one day post-passage. Cells were cultured under sterile conditions, and maintained at 37°C, 5% CO_2_.^14^

### HEK293T

HEK293T cells were grown in Dulbecco’s modified Eagle’s medium (DMEM) (Invitrogen 11995-073) supplemented with 10% fetal calf serum (FBS) (ATCC 30-2020) and maintained at 37°C, 5% CO_2_.

### Mice

Mice were housed in a pathogen-free facility at the New Research Building at Harvard Medical School, fed *ad libitum* on a 14/10h light/dark cycle and checked daily by veterinary staff. All *in vivo* experiments were carried out in accordance with the Institutional Animal Care and Use Committee (IUCAC) guidelines reviewed and approved under the IUCAC guidelines at Harvard Medical School. Adult C57BL/6N-Alpk3^tm1b(EUCOMM)Hgmu^/H were obtained from Medical Research Council Harwell and all mice were at least doubly housed. Genomic DNA was extracted and genotypes were confirmed (Figure S4) using PCR with the KOD Hot Start DNA polymerase kit using three primers (Forward primer: 5’ CTCCTTGAGGGTTAGCTGCCTT 3’, Reverse primer (WT): 5’ CCTGTGACAATGCAGGTGAACC 3’, Reverse primer (*Alpk3*-null) 5’ CTACCCCAGACCTTGGGACCA 3’), an annealing temperature 54°C, and extension time of 40 seconds for 35 cycles.

Echocardiograms were conducted at birth and periodically throughout life. Mice were sacrificed and a four-chamber cardiac dissection was performed under a dissecting microscope to obtain left ventricular (LV) tissue for subsequent analysis. The sex of all animals was determined either by visual inspection or by *Sry* PCR for P0 pups. The sex ratio of litters was balanced and both males and females were used for all experiments.

### Human Tissues

Discarded LV tissues were obtained from patients with *ALPK3* variants undergoing clinically indicated interventions using human subject research protocols, approved by Massachusetts General Brigham. Patients or parents provided written consent and assent and the study was approved by an institutional review committee. Tissues were processed for snRNAseq as described.^15^ Data from diseases hearts were compared to previously reported normal adult human LV tissues.^15^

### Multiple sequence alignment of the α-kinase domain and structural models

Clustal Omega^16^ was used for multiple sequence alignment of α-kinase domains from all human α-kinases, and their orthologs in multiple species (see UniProt accession IDs in Figure S1). Visualization and analysis of multiple sequence alignment was performed in JalView.^17^

The solved TRPM7 α-kinase domain crystal structure (Protein Data Bank (PDB) 1IA9_A, refined with Modeller)^18,19^ was used to annotate homologous ALPK3 kinase domain residues^10^ with UCSF Chimera.^20^

### CRISPR/Cas9 genome editing

All guide RNAs and homology-directed-repair arms were designed with Benchling. For the *ALPK3* exon 6 guideRNA, used to produce the *ALPK3*^ins/del^ line, a fragment bearing all components necessary for gRNA expression (U6 promoter + target sequence (AGAAGAATGTGCAGGCAGAT) + guide RNA scaffold + termination signal) was synthesized as a gBlock from Integrated DNA Technologies (IDT) and cloned into the TOPO plasmid using the Zero Blunt Topo II PCR cloning kit (Invitrogen K2800-02). A combination of 4μg of guideRNA TOPO plasmid and 2μg of Cas9 plasmid (pSpCas9(BB)-2A-Puro (PX459) V2.0 (Addgene 62988) was used for genome editing as described previously.^21^ *ALPK3* CRISPR RNAs were synthesized as custom ALT-R CRISPR crRNA from IDT. The crRNA (*ALPK3*^dIC/dIC^ targeting sequence:

CTCTCTTTTCAGGGGTTGAC; *ALPK3*^sIC/sIC^ targeting sequence:

CAGCTTCCTTGTCACAGACT; *ALPK3*^del/del^ targeting sequence:

TACCTGCCAAGTCTGTGACA) was annealed with the 5’ ATTO 550 trcRNA to create the guideRNA, which was incubated together with the ALT-R S.p HiFi Cas9 3NLS to create the function ribonucleoprotein (RNP) complex per IDT protocol. The *ALPK3* ssHDR templates were synthesized by IDT as an Ultramer ssDNA oligo (*ALPK3*^dIC/dIC^ sequence:

AGTATATCTTCCTGCTGGAGATGTGGGGGCTTAGAGCCAGCCCAGACTGGCATCAACTCCCAACTTTCTCTCTTTTCAGAAGTTGACTGGAAGATGACTGCTGTGCAGATTGCTACCAAACTCCGAGGGTGAGTGGTTCTT; *ALPK3*^sIC/sIC^ sequence:

GAGGCTCCGACAGCATCTGGCAGCTCTGAGGCCATGCAGAAATGCCAGACCTTCCAACACTGGCTGTATCAGTGGACAAATGGCAGCTTCCTTGTCACAGCCCTTGCAGGTACGAGGGTGTGAGGGTGCACGGGTACGCATGTGCATGGATGTGAAAGCATGCAGAGGAGGCAAAGCCATAGTGCTTGGCTGATCGTTTA; *ALPK3*^del/del^ mutant sequence:

GAGGCTCCGACAGCATCTGGCAGCTCTGAGGCCATGCAGAAATGCCAGACCTTCCAACACTGGCTGTATCAGTGGACAAATGGCAGCTTTCTTGTCACAGCCTTGGCAGGTACGAGGGTGTGAGGGTGCACGGGTACGCATGTGCATGGATGTGAAAGCATGCAGAGGAGGCAAAGCCATAGTGCTTGGCTGATCGTTTA).

For nucleofection of CRISPR components, adherent hiPSCs were dissociated with accutase and washed 1X with PBS. Approximately 1x10^6^ hiPSCs were resuspended in 82μL of human stem cell nucleofector solution and 18μL of supplement 1 (Lonza). CRISPR components (either guideRNA plasmid + Cas9 plasmid or CRISPR RNP complex + 1uL of 100uM ssHDR template) were added, and the mixture was transferred into an electroporation cuvette, and nucleofected using program B-016 on the Amaxa 2B nucleofector (Lonza). For plasmid-based CRISPR, puromycin (Invitrogen A11138-03) selection at a concentration of 0.5μg/mL was continued for 2 days post-nucleofection.^22^

After approximately one-week, single rounded colonies were picked under a dissection microscope using sterile pipette tips into a new Matrigel-coated 96-well plate in the presence of 5μM ROCK inhibitor Y-27632 (R&D Systems, 125410).

Genomic DNA was extracted using PrepGEM (VWR PUN0500) and colonies were screened for the presence of gene editing by PCR amplification followed by Sanger sequencing. Colonies with evidence of gene editing were subcloned (≥5 independent clones) to verify clonality. Clonality of populations containing indels were further confirmed by next generation sequencing analysis (MiSeq, Figure S2).

### Ploidy analysis using low-read depth next generation sequencing

Genomic DNA collected from hiPSCs cultured in one well of a six well plate was extracted with DNeasy Blood&Tissue kit (Qiagen, 69504) per manufacturer protocol. DNA quality and concentration were assessed by Agilent 4200 TapeStation system (Agilent Technologies, G2991BA) and 1 ng genomic DNA for each sample was tagmented with the Nextera XT DNA Library prep kit (Illumina, FC-131-1096). Libraries were amplified and cleaned with AMPure XP beads (Agencourt, A63882). Equal amounts of libraries were pooled and sequenced, targeting on at least 200,000 reads per sample and mapped to the hg38 reference genome. Bam files were loaded into R studio (version 4.0.1). Raw reads were counted with a fixed bin size of 500 using ‘*getBinAnnotations()*’ and ‘*binReadCounts ()*’ function, and then normalized to total read counts of corresponding chromosomes. For each chromosome, normalized counts from different samples were visualized in a single plot with ‘*plot()*’ function for ploidy comparisons (Supplemental PDF S5-S6).

### Differentiation of hiPSC-CMs

hiPSCs were differentiated into cardiomyocytes in monolayers as described previously with minimal modification.^23^ hiPSCs seeded in Matrigel-coated 6-well culture plates were grown to 90% confluency prior to initiating the hiPSC-CM differentiation. To initiate differentiation, 18μM CHIR-99021 (Tocris 44-231-0) in RPMI media (Invitrogen) supplemented with B27 minus insulin (Thermo Fisher Scientific) was added to hiPSCs for 1 day (day 0-1). The media was changed to RPMI with B27 minus insulin for two days (day 1-2). Cultures were then treated with 2μM Wnt-C59 (Biorbyt orb181132) in RPMI supplemented with B27 minus insulin for two days (day 3-5). Cells were maintained in RPMI with B27 minus insulin for 2 additional days (day 6-7). On day 7, media was changed to RPMI with B27 plus insulin (Invitrogen) and the media was replenished every other day until day 11, at which point cells underwent a 7-day glucose-deprivation protocol to enrich for cardiomyocytes; for enrichment, cells were cultured in RPMI media devoid of glucose (Life Technologies) supplemented with B27 with insulin, which was replaced every other day until day 17. On day 17, cells were again cultured growth media containing glucose (RPMI supplemented with B27 plus insulin) with media replacement every other day. All proteomics and RNAseq experiments were performed with hiPSC-CMs at day 30-32 of differentiation. All other assays (sarcomere contractility, immunofluorescence) were performed with hiPSC-CMs that were between day 30 to day 40 of cardiomyocyte differentiation.

### Evaluation of sarcomere contractile function in hiPSC-CMs

hiPSC-CMs were lifted and replated onto Matrigel-coated glass-bottom imaging-optimized 12-well plates (Mat-Tek) at day 20 of cardiomyocyte differentiation as previously described.^14^ hiPSC-CMs were cultured in RPMI B27 plus insulin containing 5μM ROCK inhibitor and 20% FBS for 2 days post-replating, after which they were cultured in RPMI B27 plus insulin until day 30-32 when imaging was performed. For (GFP) video microscopy, hiPSC-CMs were maintained in a heated and humidified chamber (37C, 5% CO_2_) attached to the stage of a fluorescence microscope (Keyence BZ X-710). Five-second videos of sarcomeres in beating hiPSC-CMs (typically clusters of 2-4 cells each) were acquired using a 100X oil objective at an acquisition rate of 30 frames per second. Analysis of sarcomere contraction was performed using SarcTrack software.^24^

### Transient transfection of HEK293T cells

HEK293T cells were grown in 100mm culture dishes to 80% confluency and transfected with 7.5μg total plasmid(s) DNA encoding human ALPK3-FLAG (Origene RC220076) using Lipofectamine 3000 (Invitrogen L3000001) per manufacturer protocol alongside non-transfected control dishes. Cells were harvested at 72h post-transfection for analysis.

### Harvest of cell and tissue lysate for Western blotting and mass spectrometry

Cells (HEK293T cells/hiPSC-CMs at day 30 of cardiomyocyte differentiation) were washed quickly in cold PBS. Adherent cells were scraped from tissue culture dish (100mm plate for HEK293T cells, all wells of a 6-well culture dish for hiPSC-CMs) and pelleted by spinning at 1000RPM for 5 minutes.

Cells were then lysed with 0.5-1mL cold RIPA buffer in the presence of protease inhibitors (Sigma-Aldrich 11873580001) and phosphatase inhibitors (Sigma-Aldrich 4906837001). To increase protein yield and ensure lysis of nuclei, the lysate was subjected to additional mechanical disruption by passage 20 times through a 21G needle. The mixture was shaken gently on ice for 15 minutes, and then centrifuged at >12,000g for 10-15 minutes to pellet cell debris. The supernatant was transferred to a new tube and protein concentration was quantified using the BCA assay kit (Pierce).

LV tissue from mouse/human (~2x2mm piece) was lysed in 200μL RIPA containing protease and phosphatase inhibitors as above, and subsequently homogenized in TissueLyser (Qiagen) for 5 minutes at 25Hz. Homogenized tissue was spun at 14,000g for 15min to pellet cell debris. Supernatant was transferred to a new tube and protein concentration was quantified using the BCA assay kit (Pierce).

### Coimmunoprecipitation

HEK293T cells, grown to confluence in a 10cm tissue culture dish, were transfected with 15μg total plasmid DNA (human ALPK3-FLAG: Origene RC220076, FLAG-FOXI3: Addgene Plasmid #153128, MYOM1-HA: GenScript OHu 15548, and/or MYOM2-HA: GenScript Ohu 15548C) using Lipofectamine 3000 (Invitrogen L3000001) per manufacturer protocol. FLAG-IP was performed using anti-FLAG® M2 magnetic beads per manufactuer protocol (Sigma-Aldrich M8823) and eluted with SDS-PAGE sample buffer for analysis by Western Blot.

### Western blotting

Protein lysates were loaded and resolved under denaturing conditions on a NuPAGE 3-8% Tris-Acetate gel or NuPAGE 4-20% Tris-Glycine gel. Lysate was transferred to a PVDF membrane overnight at 40mA at 4°C. 5% skim milk in 1X TBST buffer was used as a blocking agent. Primary antibodies used for Western blotting include mouse anti-FLAG 1:1000 (Sigma-Aldrich, F1804), rabbit anti-HA 1:1000 (CST, C29F4), rabbit anti-MYOM1 1:1000 (Abcam, ab201228), mouse anti-cardiac troponin T 1:500 (Abcam, ab8295), rabbit anti-MYOM2 1:500 (Abcam, ab93915), rabbit anti-TUBB 1:1000 (Abcam, ab6046), mouse anti-MuRF1 1:1000 (Santa Cruz, sc-398608), rabbit anti-CAPN3 1:1000 (Proteintech, 104-92-1-AP). Blots were blocked for at least 30 minutes at room temperature prior to addition to primary antibodies, incubated at 4°C overnight, washed 3X with TBST prior to addition of mouse or rabbit secondary antibodies (Abcam ab97023, ab205718). Secondary antibodies were applied for 1-2 hours at 1:5000, washed 3X with TBST, and detected using Clarity Western ECL chemiluminescent substrates per manufacturer protocol (Bio-Rad, 1705060).

### Tandem-Mass-Tag proteomics and phosphoproteomics

Protein extracts were prepared and subjected to streamlined Tandem Mass Tag (SL-TMT) quantitative (phospho)proteome profiling using synchronous precursor selection-MS3 as described previously^25^. For experiments with hiPSC-CMs, multiple TMT experiments were run: one phosphoproteomics 11-plex (WT n=4, *ALPK3*^dIC/dIC^ n=4, *ALPK3*^ins/del^ n=3) from which both phosphopeptide and protein abundances were determined, and another 10-plex with additional differentiations of hiPSC-CMs (WT n=4, *ALPK3*^dIC/dIC^ n=3, *ALPK3*^ins/del^ n=3) from which protein abundances were determined and normalized to WT levels for comparison across both 10- and 11-plex experiments (5,587 total proteins, 3712 phosphopeptides quantitated in both experiments). To later validate results, another phophoproteomics 12-plex with additional cell lines was performed (WT n=4, *ALPK3*^sIC/sIC^ n=4, *ALPK3*^del/del^ validation n=4; 7,970 total proteins, 5,818 phosphopeptides).

Briefly, cells/tissues were lysed, after which cysteine bonds were reduced with 5mM tris(2-carboxyethyl)phosphine (TCEP) and alkylated with 10mM iodoacetamide that was quenched with 10mM DTT. A total of 100μg of protein per sample methanol-chloroform precipitated to extract proteins, which were subsequently digested using Lys-C (Wako) overnight followed by digestion with trypsin (ThermoFisherScientific) for 6h. The resulting peptides were labeled with tandem-mass-tag (TMT) isobaric tags. To check mixing ratios, 2μg of each sample was pooled, desalted, and analyzed by mass-spectrometry. Using normalization factors calculated from this “label check”, samples were mixed 1:1 across all channels, and a single desalting step was performed using a 100mg Sep-Pak solid-phase extraction column (Waters). The dried, mixed, and desalted sample was subjected to centrifugation-based phosphopeptide enrichment using the Pierce High-Select Fe-NTA phosphopeptide enrichment kit (ThermoFisher Scientific). Enriched phosphopeptides were desalted for SPS-MS3 analysis. The flow-through unbound fraction and washes from this enrichment were combined, desalted, and fractionated by basic pH reverse-phase HPLC. The resulting fractions were desalted by StageTip and analyzed by liquid chromatography-tandem mass spectrometry (LC-MS/MS). Mass spectrometric data were collected on an Orbitrap Fusion mass spectrometer in line with a Proxeon NanoLC-1200 UHPLC. Database searching and reporter-ion quantification was performed using an in-house SEQUEST-based pipeline. Each protein was scaled such that the summed signal-to-noise for that protein across all channels was 100, thereby generating a relative abundance measurement.

### RNA sequencing

hiPSC-CMs and mouse left ventricular tissue were lysed in Trizol Reagent (Life Technologies); mouse tissue was subjected to additional two minutes of mechanical bead homogenization using a TissueLyser (Qiagen). RNAseq was performed as previously described ^26^. Briefly, RNA was isolated by chloroform:isopropyl alcohol extraction. RNA quality (RIN) and quantity were assessed on the TapeStation 2200 (Agilent). Two rounds of mRNA purification were performed on total RNA (1 ug) using Dynabeads mRNA DIRECT Kit (Invitrogen). The Superscript III First-Strand Synthesis System (Invitrogen) was used to generate double-stranded cDNA. cDNA libraries were constructed using the Nextera XT DNA Library Preparation Kit (Illumina). Libraries were sequenced on the Illumina NextSeq500 platform. 75 bp paired-end reads were aligned to the human reference genome hg38 using Spliced Transcripts Alignment to a Reference (STAR). Raw reads were normalized to the total number of reads per kilobase of transcript per million (RPKM). For analysis of human left ventricular tissues, nuclei were isolated and single nuclear RNA sequencing was performed as described elsewhere.^15,27^

### Mouse echocardiography

Cardiac function was evaluated by a skilled echocardiographer who was blinded to mouse genotypes. Newborn pups were not anesthetized, while older mice were anesthetized under isoflurane vaporizer (VetEquip). Each limb was placed on the ECG leads on a Vevo Mouse Handling Table (FujiFilm VisualSonics) to maintain body temperature at 37°C during the study. Chest hair was removed with depilatory cream to obtain clear images. Anesthesia was terminated after a mouse was properly positioned for imaging, and all measurements were performed with a heart rate between 300 and 550 beats per minute. Two-dimensional and M-mode images of the left ventricle (parasternal long axis and short axis) were obtained. Measurements were averaged from M-mode tracings of 3 consecutive heart beats including left ventricular end-diastolic dimension (LVEDD), end-systolic dimension (LVESD), left-ventricular posterior wall thickness (LVPW), interventricular septal thickness (IVS). Left ventricular fractional shortening was calculated by this equation: FS = 100 x [(LVEDD-LVESD)/LVEDD].

### Immunofluorescence

hiPSC-CMs were replated onto Matrigel-coated glass-bottom imaging-optimized 12-well plates (Mat-Tek) ^28^. hiPSC-CMs were cultured in RPMI B27 plus insulin containing 5μM ROCK inhibitor and 20% FBS for 2 days post-replating, after which they were cultured in RPMI B27 plus insulin for 1 week prior to imaging analysis. For transient transfection experiments, replated hiPSC-CMs were transfected with 1μg of total plasmid DNA encoding human ALPK3-FLAG (Origene RC220076), MYOM1-HA (GenScript OHu 15548), and/or MYOM2-HA (GenScript OHu 15548C) using Lipofectamine 3000 (Invitrogen L3000001) per manufacturer protocol and incubated for 72hrs prior to imaging.

Murine cardiomyocytes were isolated from 7-9-week old mice by rapid excision of the heart and aortic cannulation on a Langendorff apparatus as described previously.^29^ Isolated cardiomyocytes were plated in glass-bottom imaging-optimized 12-well plates (Mat-Tek) that had been precoated with laminin at a concentration of 10μg/ml in phosphate-buffered saline (PBS).^29^

hiPSC-CMs or murine cardiomyocytes were fixed with 4% PFA (10 min), rinsed with PBS (5 min x3), permeabilized with 0.2% Triton-100 (5 min), rinsed with PBS 1x, and incubated with primary antibody overnight at 4°C in 3% BSA in PBS (mouse anti-FLAG M2, Sigma-Aldrich F1804 1:1000; rabbit anti-HA, Cell Signaling Technologies C29F4 1:000), rabbit MYOM2, abcam ab93915 1:100 and mouse MYOM1 (distributed as mMaC myomesin B4) 0.3μg/mL, Developmental Studies Hybridoma Bank). Wells with rinsed with PBS (5 min x3) and samples were incubated with respective secondary antibodies (abcam ab150080, ab150116, abcam 150077, abcam ab150113) at a dilution of 1:1000 for 2 hr at room temperature prior to imaging with a spinning disk confocal microscope (Yokogawa CSU-W1 Spinning Disk on Nikon T1). Images were analyzed using Fiji software.

### Electron Microscopy

Mice were sacrificed and left ventricular tissue was isolated under a dissecting microscope. Left ventricular tissue was cut into small pieces (1-2mm cubes) and perfused in 1% glutaraldehyde/3% paraformaldehyde in cardioplegic buffer (5% dextrose, 30mmol/L KCl in PBS).^30^ After fixation the tissue was washed in 0.1M cacodylate buffer and postfixed with 1% Osmiumtetroxide (OsO4)/1.5% Potassium ferrocyanide (KFeCN6) for 1 hour, washed in water 3x and incubated in 1% aqueous uranyl acetate for 1hr followed by 2 washes in water and subsequent dehydration in grades of alcohol (10min each; 50%, 70%, 90%, 2x10min 100%). The samples were then put in propyleneoxide for 1 hr and infiltrated ON in a 1:1 mixture of propyleneoxide and TAAB Epon (Marivac Canada Inc. St. Laurent, Canada). The following day the samples were embedded in TAAB Epon and polymerized at 60°C for 48 hrs. Ultrathin sections (about 80nm) were cut on a Reichert Ultracut-S microtome, picked up on to copper grids, stained with lead citrate and examined in a JEOL 1200EX Transmission electron microscope or a TecnaiG^2^ Spirit BioTWIN. Images were recorded with an AMT 2k CCD camera and saved as TIFF files.

### Quantification and Statistical Analysis

All statistical comparisons and sample sizes are included in the Figures and Figure Legends. Unpaired two-sided t-tests were used to determine p-values for all proteomics/phosphoproteomics analysis. DESeq2 was used to analyze RNA sequencing data and determine differentially expressed RNAs^31^. No samples were excluded. Data were visualized and graphics were generated using the RStudio programming environment, Prism 8 (GraphPad) and Biorender. Non-linear lines-of-best-fit for mouse echocardiography parameters of cardiac function were fitted using Prism 8. In experiments using cell lines, “n” indicates independent differentiations into hiPSC-CMs. In experiments using mouse tissue, “n” refers to number of animals.

## Results

### Structure and conservation of the ALPK3 α-kinase domain

We examined the relative conservation of the ALPK3 α-kinase domain by sequence alignment relative to the five other human α-kinase protein domains (Figure 1B). This analysis identified sixteen amino acid residues that are entirely conserved amongst all human α-kinase family members (Figure 1B; red). Four additional residues are conserved in all α-kinases except ALPK3 (Figure 1B; green). Moreover, these four residues are conserved in all α-kinase orthologues (mouse, chicken, frog, zebrafish, and the evolutionarily distant slime mold *dictyostelium discoideum*) except for the ALPK3 orthologues (Figure 1B, Figure S1). We denote these four as ALPK3-specific nonconserved residues.

As the structure of ALPK3 is unsolved, we mapped the corresponding ALPK3 residues onto the TRPM7 α-kinase domain by sequence homology (Figure 1C, Table S1).^10,19^ The sixteen conserved residues were found to reside within the P-loop, interlobe cleft region which positions ATP, and C-lobe region which chelates zinc and stabilizes the tertiary domain structure^10,19^ (Figure 1C, Table S1). All four ALPK3-specific nonconserved residues were contained in the interlobe cleft, two of which (S1731, A1776) occupied positions involved in positioning ATP and one of which (S1800) is predicted be involved in substrate binding.^10,11^ The absence of these four residues in ALPK3 that are otherwise entirely conserved amongst all α-kinase family members prompted the hypothesis that ALPK3 might lack catalytic activity.

### Characterization of *ALPK3* mutant hiPSCs and differentiation into hiPSC-CMs

To investigate the putative catalytic activity of ALPK3, we used CRISPR/Cas9 gene editing to create isogenic human induced pluripotent stem cells (hiPSCs) carrying either wild-type (WT) or mutated *ALPK3* alleles and then differentiated these into hiPSC-derived cardiomyocytes (hiPSC-CMs). These hiPSCs also contained an endogenous N-terminal green fluorescent protein (eGFP) tag on titin (TTN), a protein spanning the Z-disc to M-band of sarcomeres, that enables monitoring of sarcomere contraction in hiPSC-CMs.^14,24^

The *ALPK3*^ins/del^ hiPSC line carries compound frameshift variants (Table 1). A single base insertion (c.2442_2443insA) causes a missense residue at position p.815 and results in a premature stop codon; the other allele contains a deletion (c.2428_2458del31) that excises residues p.809-819 and results in a premature stop codon. The *ALPK3*^G1777ED1784A/G1777ED1784A^ hiPSC line (henceforth denoted as ‘dual interlobe cleft’ by *ALPK3*^dIC/dIC^) carries homozygous dinucleotide (c.5330-5331GG>AA) and mononucleotide (c.5351A>C) substitutions. These substitutions alter two conserved residues which reside in the kinase domain interlobe cleft in close proximity to the ATP binding pocket (G1777E and D1784A; Figure 1C, Table 1) and are predicted to alter the conformation of the α-kinase domain.

We also produced two additional *ALPK3* mutant lines to further study and validate results (Table 1). The *ALPK3*^del/del^ hiPSC line carries compound frameshift variants. One allele contains a 20-base deletion (c.5306_5325del20) that produces a missense residue at p.1769, excises residues p.1770-1775 and causes a premature stop codon; the other allele contains an 11-base deletion (c.5310_5320del11) that produces a missense residue at p.1770, excises residues p.1771-1773 and causes a premature stop codon. The *ALPK3*^D1774A/D1774A^ hiPSC line (henceforth denoted as ‘single interlobe cleft’ by *ALPK3*^sIC/sIC^) carries homozygous mononucleotide (c.5321A>C) substitutions that alter a conserved aspartate residue (predicted to be the essential “catalytic” aspartate) in the kinase domain (D1774A; Figure 1C, Table 1). All hiPSC lines were subcloned, sequence validated (Figure S2A-D) and ploidy confirmed across all chromosomes (Methods).

As prior reports suggested that *ALPK3* expression might affect cardiomyocyte maturity,^12^ we compared the differentiation potential of WT and *ALPK3*^ins/del^ hiPSCs. Both WT and *ALPK3*^ins/del^ hiPSC differentiations produced beating hiPSC-CMs with similar contractile performance (Methods, see below). Principal component analyses of RNAseq at four timepoints over the 30-day differentiation protocol confirmed comparable maturation efficiency of WT and *ALPK3*^ins/del^ hiPSC-CMs (Figure S2E), with similar transcript expression of prototypic cardiomyocyte genes by day 30 (Supplementary Data S1).

To investigate the putative kinase activity of ALPK3, multiple independent batches of WT (n=8), *ALPK3*^dIC/dIC^ (n=4), and *ALPK3*^ins/del^ (n=7) hiPSCs were differentiated into hiPSC-CMs and the resultant protein extracts were analyzed by multiplexed mass spectrometry using tandem-mass-tag phosphoproteomics (Methods) with 3,714 phosphopeptides (Supplementary Data S2) and 5,587 proteins (Supplementary Data S3) quantified in all samples. *ALPK3*^ins/del^ hiPSC-CMs had profoundly reduced ALPK3 protein expression (<5% WT), with reduced levels of ALPK3 peptides across the entire length of the protein (Figure S2F). Unexpectedly, *ALPK3*^dIC/dIC^ hiPSC-CMs had approximately twice ALPK3 protein levels as WT (Figure S2F), though there was no increase in *ALPK3* transcript levels, as assessed by RNAseq (Figure S2G). We suggest that *ALPK3*^dIC/dIC^ hiPSCs, which contain substitutions at two conserved amino acid residues, may alter the kinase domain conformation and inhibit interactions with ubiquitinases or other degradatory machinery, and thereby increase protein levels. *ALPK3*^sIC/sIC^ hiPSC-CMs had comparable levels of ALPK3 protein as WT (Figure S2H). *ALPK3*^del/del^ hiPSC-CMs had reduced ALPK3 protein expression (~39% WT), indicating that these cells produce a truncated ALPK3 protein with reduced stability (Figure S2H).

### ALPK3 is a pseudokinase which lacks significant catalytic activity

To investigate the putative catalytic activity of ALPK3, we initially compared the phosphoproteomes of WT, *ALPK3*^dICd/IC^, and *ALPK3*^ins/del^ hiPSC-CMs. We hypothesized that if ALPK3 were a catalytically active kinase, its substrates would have depleted phosphorylation in both the *ALPK3*^dIC/dIC^ and *ALPK3*^ins/del^ lines.

**Figure 2:**
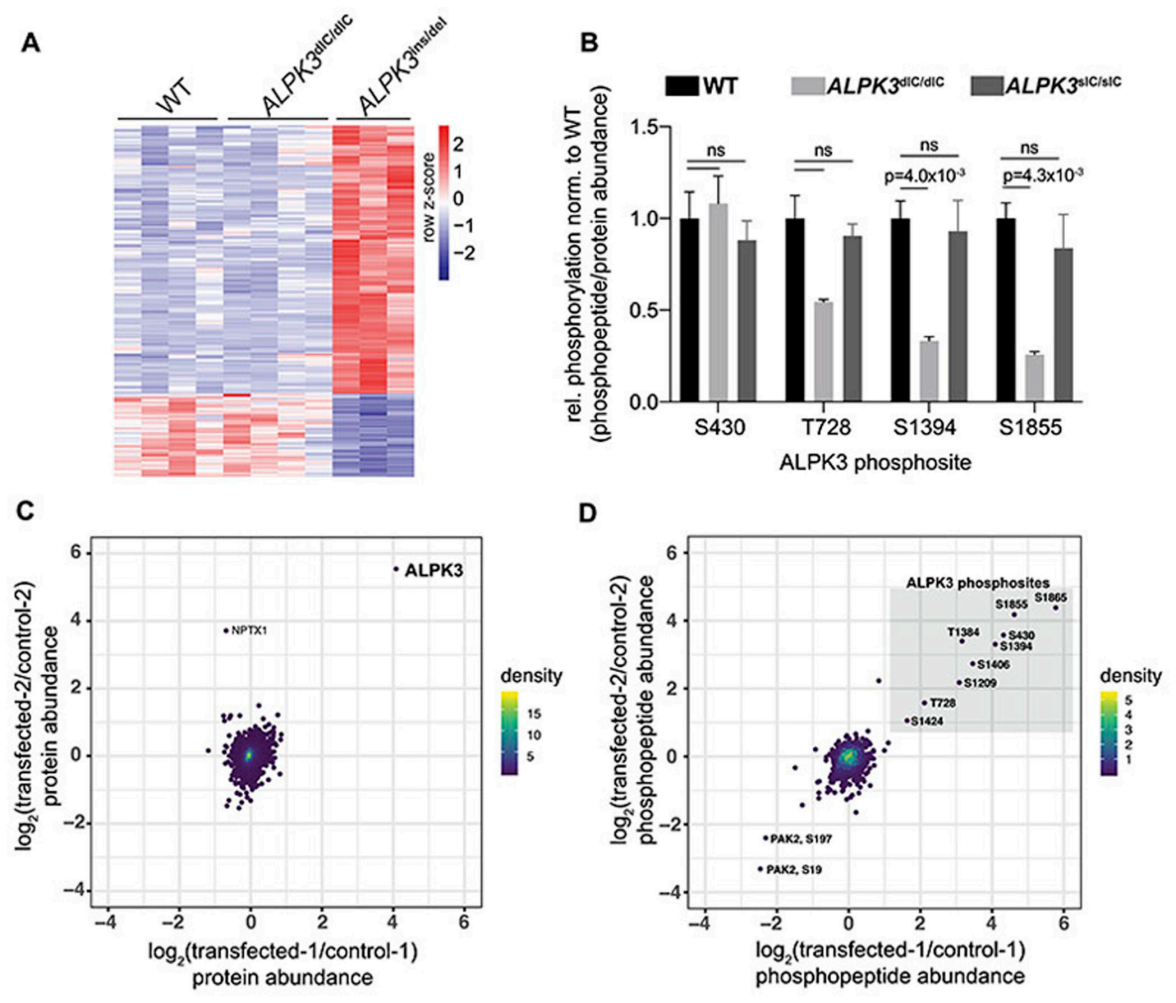
Phosphoproteomic analyses of ALPK3 kinase domain inhibition and overexpression fail to detect ALPK3 catalytic activity. **(A)** Heatmap of differentially phosphorylated (DP) sites in *ALPK3*-mutant hiPSC-CMs versus WT determined by tandem mass tag phosphoproteomics. 3,714 total phosphosites were identified on 1,630 unique proteins (see also Supplementary Data S2); Columns represent independent cultures of each hiPSC cell genotype (see Methods). In comparison to isogenic WT, *ALPK3*^ins/del^ hiPSC-CMs have 225 DP sites (171 up, 54 down) and *ALPK3*^dIC/dIC^ hiPSC-CMs have 3 DP sites (1 up, 2 down). DP criteria: |log_2_-fold change| > 0.67 & unadjusted p-value < 0.01, two-sided t-test (WT n=4; *ALPK3*^dIC/dIC^ n=4; *ALPK3*^ins/del^ n=3). **(B)** Four phosphosites are detected on the ALPK3 protein. The (only) two DP sites with reduced phosphorylation relative to protein expression in *ALPK3*^dIC/dIC^ hiPSC-CMs occur on ALPK3 S1394 and ALPK3 S1855; these sites are not affected in *ALPK3*^sIC/sIC^ hiPSC-CMs (n=4). **(C)** Log-log plot of protein abundance in HEK293T cells transfected with ALPK3 versus control cells. Each point represents a single protein (n=6,596). The level of ALPK3 in transfected samples is highly increased relative to controls (see also Supplementary Data S4, ALPK3 transfected n=2; control n=2). **(D)** Log-log plot of phosphopeptide abundance (n=1,158 phosphopeptides) in HEK293T cells transfected with ALPK3 versus control cells. The levels of nine ALPK3 phosphopeptides (grey box, phosphorylated residues denoted) were highly increased in ALPK3-transfected versus control cells, while all other phosphopeptides were minimally affected (see also Supplementary Data S4, ALPK3-transfected n=2; control n=2). For C-D, point density is indicated by color scale as shown.

Analyses of 3,714 phosphopeptides revealed that the patterns of phosphorylation of *ALPK3*^dIC/dIC^ and WT hiPSC-CMs were nearly identical (Figure 2A). Only two sites had significantly depleted phosphorylation in *ALPK3*^dIC/dIC^ hiPSC-CMs relative to WT, both of which resided within the ALPK3 protein itself (ALPK3 residues S1384 and S1855) (Figure 2B, Supplementary Data S2). While *ALPK3*^ins/del^ hiPSC-CMs had many more differentially phosphorylated sites (n=225), approximately 75% of these had *increased* phosphorylation relative to WT (Figure 2A, Supplementary Data S2), indicating that these did not represent direct ALPK3 substrates.

Additional phosphoproteomic investigations of *ALPK3*^sIC/sIC^ hiPSC-CMs confirmed that this line had very few differences in phosphorylation relative to WT (n=19 differentially phosphorylated peptides vs. expected ~50 false positives (calculated as 0.01x5009 total phosphopeptides), and no differences in phosphorylation on sites residing within the ALPK3 protein relative to WT (Figure 2B, Supplementary Data S2). These findings suggested that the altered phosphorylation on ALPK3 S1384 and S1855 in *ALPK3*^dIC/dIC^ hiPSC-CMs is likely due to the altered conformation of the kinase domain in this mutant rather than loss of any auto-phosphorylative activity. Analyses of differentially phosphorylated sites in *ALPK3*^del/del^ hiPSC-CMs showed few differentially phosphorylated sites relative to *ALPK3* ^ins/del^ hiPSC-CMs (n=15 vs. 225, Supplementary Data S2). These findings suggest that *ALPK3*^del/del^ hiPSC-CMs produce an unstable truncated ALPK3 protein (Figure S2H) which still may be able to participate in noncatalytic (i.e. scaffolding) roles, unlike ALPK3^ins/del^ hiPSC-CMs which have near-complete loss of ALPK3 protein expression (Figure S2F). Based on these data and the paucity of differentially phosphorylated sites in both *ALPK3*^dIC/dIC^ and *ALPK3*^sIC/sIC^ hiPSC-CMs we inferred that ALPK3 lacks significant catalytic activity.

We confirmed the absence of ALPK3 catalytic activity by transiently overexpressing *ALPK3* in HEK293T cells, and comparing the phosphoproteomes (1,158 unique phosphopeptides, 6,596 unique proteins) of transfected and control cells (Supplementary Data S4). ALPK3 protein expression was >16-fold higher in transfected versus control cells (Figure 2C, Figure S3A-B). Only nine phosphopeptides had reproducibly increased abundance in transfected versus control cells, and all were within ALPK3 (Figure 2D). The increased abundance of these phosphopeptides is consistent with increased ALPK3 protein abundance in the transfected lines; ALPK3 overexpression did *not* increase the phosphorylation of any of the other 1000+ phosphosites across the HEK293T phosphoproteome, supporting the conclusion that ALPK3 is a pseudokinase.

### ALPK3 loss-of-function dysregulates myomesins and associated thick filament proteins in hiPSC-CMs

**Figure 3:**
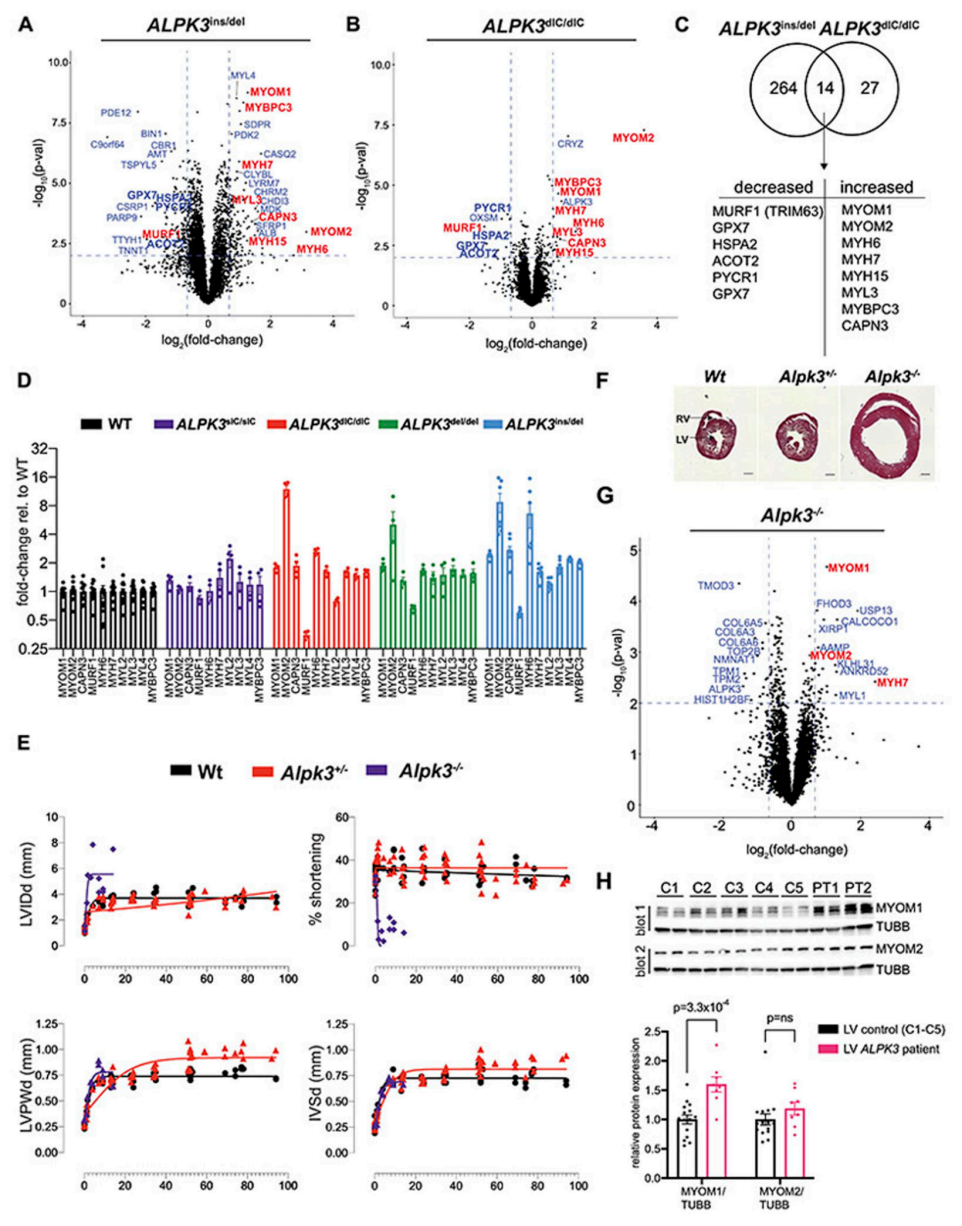
*ALPK3* variants dysregulate myomesin and thick filament proteins in hiPSC-CMs, mice, and human tissues. **(A-B)** Volcano plots of protein expression in *ALPK3*^ins/del^ hiPSC-CMs (n=7) vs WT (n=8) and *ALPK3*^dIC/dIC^ hiPSC-CMs (n=4) vs WT (n=5,587 total proteins). Dashed lines indicate cutoffs for differential expression: |log_2_-fold change| ≥ 0.67 & unadjusted t-test p-value < 0.01. Sarcomere M-band and thick filament proteins are highlighted in red (see also Supplementary Data S3). **(C)** Overlap of proteins with altered expression in *ALPK3*^ins/del^ and *ALPK3*^dIC/dIC^ hiPSC-CMs. **(D)** Fold-change of M-band and thick filament proteins in *ALPK3*^sIC/sIC^ (n=4), *ALPK3*^dIC/dIC^ (n=4), *ALPK3*^del/del^ (n=4), and *ALPK3*^ins/del^ (n=7) hiPSC-CMs relative to WT (n=12). **(E)** Serial echocardiographic measurements (x-axis, age in weeks) of Wt and *Alpk3* mutant mice. No measurements are reported in *Alpk3*^-/-^ mice after 14 weeks due to early lethality. (LVIDd: LV internal dimension at end diastole; LVPWd: LV posterior wall thickness at end-diastole; IVSd: interventricular septal thickness at end-diastole). **(F)** Transverse cardiac sections showing ventricular enlargement of *Alpk3*^-/-^ heart at 14-weeks (Masson trichrome stain, scale bar = 1mm). **(G)** Volcano plot of protein expression in LV tissues harvested at postnatal day 8 from *Alpk3*^-/-^ vs Wt mice (n=5,678 total proteins; see also Supplementary Data S3). Proteins are identified by their human homologues. Dashed lines and labels are as in (a-b). (Wt, n=4; *Alpk3*^+/-^, n=3, *Alpk3*^-/-^, n=3). **(H)** (UPPER) Western blotting analysis of LV tissues from human subjects with compound heterozygous variants in *ALPK3* (PT1-PT2) compared to adult healthy control tissues (C1-C5) probed with (blot 1) myomesin-1 (MYOM1, blot 1), tubulin (TUBB), and myomesin-2 (MYOM2, blot 2) antibodies (n=3 technical replicates per sample, see also Table S2). (LOWER) Western blotting quantification of MYOM1 and MYOM2 expression in human ALPK3-deficient subjects and ALPK3-normal subjects (normalized to TUBB levels). Data are mean±SEM.

To explore other possible functions of ALPK3, we first compared the proteomes of *ALPK3*^ins/del^ and WT hiPSC-CMs (n=5,587 total proteins). *ALPK3*^ins/del^ hiPSC-CMs had 264 differentially expressed (DE) proteins and *ALPK3*^dIC/dIC^ hiPSC-CMs had 27 DE proteins relative to WT (Figure 3A-B, Supplementary Data S3). Fourteen DE proteins were shared by both *ALPK3*^ins/del^ and *ALPK3*^dIC/dIC^ hiPSC-CMs which is significantly more than expected by chance (p=2.3x10^-12^, hypergeometric test), indicating that the *ALPK3*^dIC/dIC^ variant had partial loss-of-function effects (Figure 3C).

Several of the shared DE proteins were components of the M-band, a central transverse zone that demarcates half a sarcomere^32^ (Figure 3A-C). The major constituents of the M-band are myomesin proteins which crosslink myosins and buffer forces generated by opposing thick filaments during sarcomere contraction via multiple extensible α-helical domains.^33,34^ The levels of both myomesin-1 (MYOM1) and myomesin-2 (MYOM2, also called M-protein) were increased in *ALPK3*^ins/del^ and *ALPK3*^dIC/dIC^ hiPSC-CMs, as were the levels of thick filament proteins bound by myomesins including α-myosin heavy chain (MYH6), β-myosin heavy chain (MYH7), myosin essential light chain (MYL3), and myosin binding protein c (MYBPC3; Figure 3A-C). As myosin heavy chain isoforms and myomesins share 93% and 43% sequence identity, respectively, the abundances of these proteins were recalculated after restricting the analysis to include only uniquely mapping peptides, which confirmed that both myosin heavy chain proteins (MYH6, MYH7) and myomesin proteins (MYOM1, MYOM2) were increased in *ALPK3*^ins/del^ and *ALPK3*^dIC/dIC^ hiPSC-CMs relative to WT (Figure S5B, Supplementary Data S3). Two shared DE proteins (MuRF1, decreased; CAPN3, increased) also localize to the M-band and are known to regulate sarcomere protein turnover.^35–38^

To further study and validate these changes, we performed additional phophoproteomic studies of *ALPK3*^sIC/sIC^ and *ALPK3*^del/del^ hiPSC-CMs (Supplementary Data S2-S3). *ALPK3*^del/del^ hiPSC-CMs had similar but less severe changes in M-band and thick filament proteins levels as *ALPK3*^ins/del^ hiPSC-CMs, including increased MYOM1, MYOM2, CAPN3 and reduced MuRF1 levels (Figure 3D). Unlike *ALPK3*^dIC/dIC^, *ALPK3*^ins/del^, and *ALPK3*^del/del^ hiPSC-CMs, the *ALPK3*^sIC/sIC^ hiPSC-CMs did not show changes in myomesin and thick filament protein levels (Figure 3D), further supporting a non-catalytic role of ALPK3 in regulating the M-band and thick filament proteins.

High-throughput analyses of the sarcomere contractility (>40,000 total tracked sarcomeres per cell line) assessed using the SarcTrack algorithm^24^ did not detect significant differences between WT, *ALPK3*^ins/del^, and *ALPK3*^dIC/dIC^ hiPSC-CMs (Figure S5A).

### Alpk3 deficiency in mice causes cardiomyopathy and dysregulates myomesin and associated thick-filament proteins

As we did not detect differences in sarcomere contractility between WT and *ALPK3* mutant hiPSC-CMs, which may reflect the absence of hemodynamic influences, we assessed the *in vivo* consequences of *ALPK3* variants by studying a mammalian model. A previous *Alpk3*^-/-^ C57BL/6J mouse model,^13^ failed to recapitulate the rapidly progressive and often fatal neonatal disease is observed in human patients.^3,6^ We therefore acquired and studied an alternative model, C57BL/6N *Alpk3*^+/-^ mice (MRC Harwell Institute) that was created by deleting a region encompassing exon 3 (Figure S4). Heterozygous matings produced *Alpk3*^-/-^ mice at expected Mendelian ratios.

Serial longitudinal echocardiography beginning at postnatal day 0 (P0) showed that *Alpk3*^-/-^ mice had normal cardiac function at birth but manifested a rapidly progressive cardiomyopathy in the first week of life that resulted in premature death (no survivors beyond 14 weeks). Longitudinal cardiac imaging showed that relative to Wt, *Alpk3*^-/-^ mice had increased left ventricular (LV) cavity dimensions and severely diminished contractile function (Figure 3E-F, mean % shortening age 1-14 weeks: *Alpk3*^-/-^ (n=11) = 7.7%; Wt (n=22) = 37.5%, p=1.1x10^-9^).

*Alpk3*^+/-^ heterozygote mice had normal contractile function, fertility, and survival, but developed significant ventricular hypertrophy beyond one year of age, supporting the pathogenicity of *Alpk3* heterozygote variants in adult human patients (Figure 3E, mean LV posterior wall (LVPW) thickness age 51-90 weeks: *Alpk3*^+/-^ (n=20) = 0.95mm vs. Wt (n=16) =0.77mm, p=7.0x10^-10^).

Proteomic analyses of murine LV tissues at postnatal day 8 (n=5,789 proteins) identified no differences between *Alpk3*^+/-^ and Wt (0 DE proteins), while *Alpk3*^-/-^ had 38 DE proteins (Supplementary Data S3). Remarkably, and similar to *ALPK3*^del/ins^, *ALPK3*^del/del^, and *ALPK3*^dIC/dIC^ hiPSC-CMs, the most significant DE proteins in mice included MYOM1, MYOM2, and MYH7 (Figure 3G), which had increased levels in *Alpk3*^-/-^ relative to Wt LV tissues. Western blotting further confirmed that both splice isoforms of MYOM1 were increased in *Alpk3*^-/-^ LV tissues compared to Wt (Figure S5C). Western blots identified no significant differences in MuRF1 levels in *Alpk3*^-/-^ mouse LV tissues relative to Wt, while CAPN3 levels were increased (Figure S6, 1.8-fold increase, p=4.3x10^-2^).

Phosphoproteomic studies of mouse LV tissues (n=3,165 phosphopeptides with corresponding protein levels) showed no significant differences in phosphorylation between *Alpk3*^+/-^ and Wt and minimal differences (n=38 differentially phosphorylated peptides, 21 with reduced phosphorylation) between *Alpk3*^-/-^ and Wt tissues (Supplementary Data S2). None of these differentially phosphorylated sites in *Alpk3*^-/-^ mouse LV tissues were shared with *ALPK3*^ins/del^ or *ALPK3*^del/del^ hiPSC-CMs, as might be expected if ALPK3 were a catalytically active kinase with specific substrates (Supplementary Data S2). These findings suggested that the changes in phosphorylation in the mouse *Alpk3*^-/-^ LV tissues likely reflect indirect changes in phosphorylation secondary to loss of ALPK3 protein expression rather than loss of ALPK3 catalytic activity.

### Analysis of myomesin protein expression in *ALPK3* human patient tissues

Having demonstrated that *ALPK3* loss-of-function dysregulated myomesin proteins in both hiPSC-CMs and mice, we examined myomesin protein expression in LV tissues from two human patients with *ALPK3* compound heterozygous variants compared to five healthy adult LV tissues (genotypes and clinical characteristics summarized in Table S2). Western blot analyses revealed significant increases in MYOM1 in *ALPK3* patient tissues relative to controls (Figure 3H, 1.6-fold increase, p=3.3x10^-4^). MYOM2 levels appeared slightly increased but did not meet the threshold for statistical significance (Figure 3H, 1.2-fold increase, p=0.24). MuRF1 levels were decreased in one of the two patient tissues (derived from an adult donor with significant hypertrophy) and CAPN3 levels were increased in both tissues (Figure S6). However, due to the large variability in expression of these proteins amongst controls and limited sample size, these changes were not statistically significant, precluding definitive conclusions.

### Analysis of myomesin and thick filament transcripts in *ALPK3* mutant cardiomyocytes and LV tissues

To assess whether there were also changes in myomesin, myosin heavy chain and other sarcomere M-band protein transcripts, we analyzed bulk RNAseq from WT and hiPSC-CMs and murine LVs (Table S3 and Supplementary Data S1) and single nuclear RNAseq (snRNAseq) from *ALPK3* patient and control LVs (Table S4). In each distinct sample type (hiPSC-CMs, murine LV tissues, human LV tissues), *MYOM1* and *MYOM2* transcripts were increased 1.4 to 1.8-fold in *ALPK3* mutant versus the respective wild-type controls, but were not statistically significant in the two human samples (Table S3-S4). The expression of myosin heavy chains was heterogenous. In *ALPK3*^ins/del^ hiPSC-CMs, *MYH7* transcripts were unchanged while *MYH6* transcripts were ~4-fold increased, while in mice and human LV tissues, *MYH7* transcripts were 1.2 to 2-fold increased and *MYH6* transcripts were 1.2 to 2-fold decreased (Table S3-S4). From these analyses we concluded that gene transcription may partially contribute to the increase in myomesin and myosin heavy chain protein levels in *ALPK3* mutant cells and tissues.

### *ALPK3* variants cause myomesin accumulation and disrupt cardiomyocyte sarcomere structure

**Figure 4:**
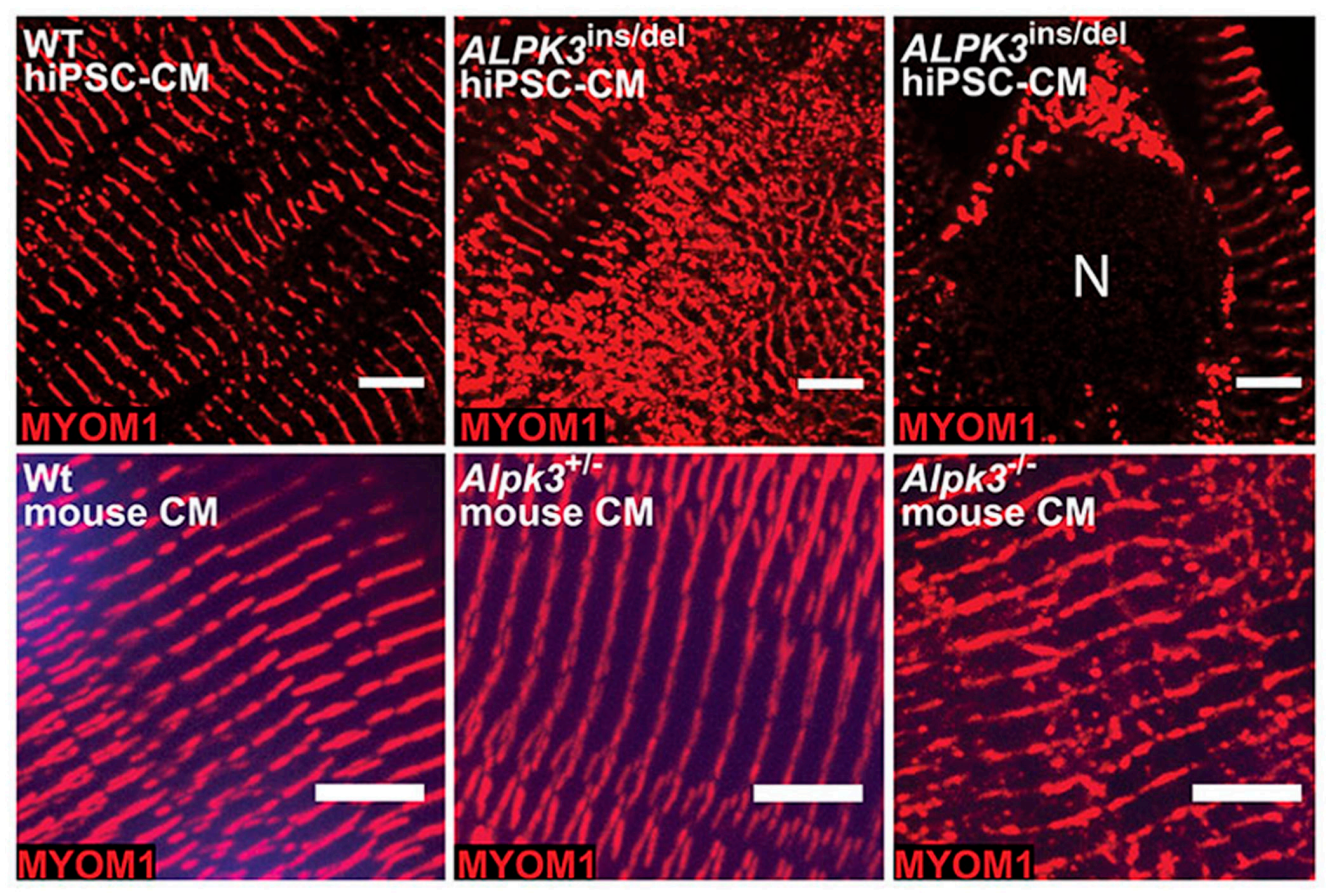
Confocal micrographs of WT and *ALPK3*-deficient hiPSC-CMs and murine cardiomyocytes stained with MYOM1 antibody (red) demonstrate myomesin-1 accumulation in mutant cells. Top row: MYOM1 accumulates outside the M-band and around the nucleus (N) in *ALPK3*^ins/del^ hiPSC-CMs versus WT. Scale bar = 5μm. Bottom row: Myom1 accumulates outside the M-band in *Alpk3*^-/-^ mouse cardiomyocytes (left ventricular cardiomyocytes isolated from mice age 7-9 weeks). Scale bar = 5μm. (See also Figure S7 for confocal micrographs of entire cardiomyocytes).

We used immunofluorescence to examine the localization and abundance of myomesins and ALPK3 in hiPSC-CMs and murine cardiomyocytes. As expected from its sarcomere interactions, MYOM1 staining was detected predominantly at the M-band in WT hiPSC-CMs (Figure 4, Figure S7). By contrast, *ALPK3*^ins/del^ hiPSC-CMs had more intense M-band staining of MYOM1 in addition to cytoplasmic foci of MYOM1 staining outside the sarcomere and surrounding the nuclear envelope, suggesting protein accumulation (Figure 4, Figure S7). Similar findings were observed in primary ventricular cardiomyocytes isolated from Wt and *Alpk3*^-/-^ mice (age 7-9 weeks) in which staining in mutant cardiomyocytes identified non-sarcomeric cytoplasmic foci of Myom1 (Figure 4, Figure S7).

**Figure 5:**
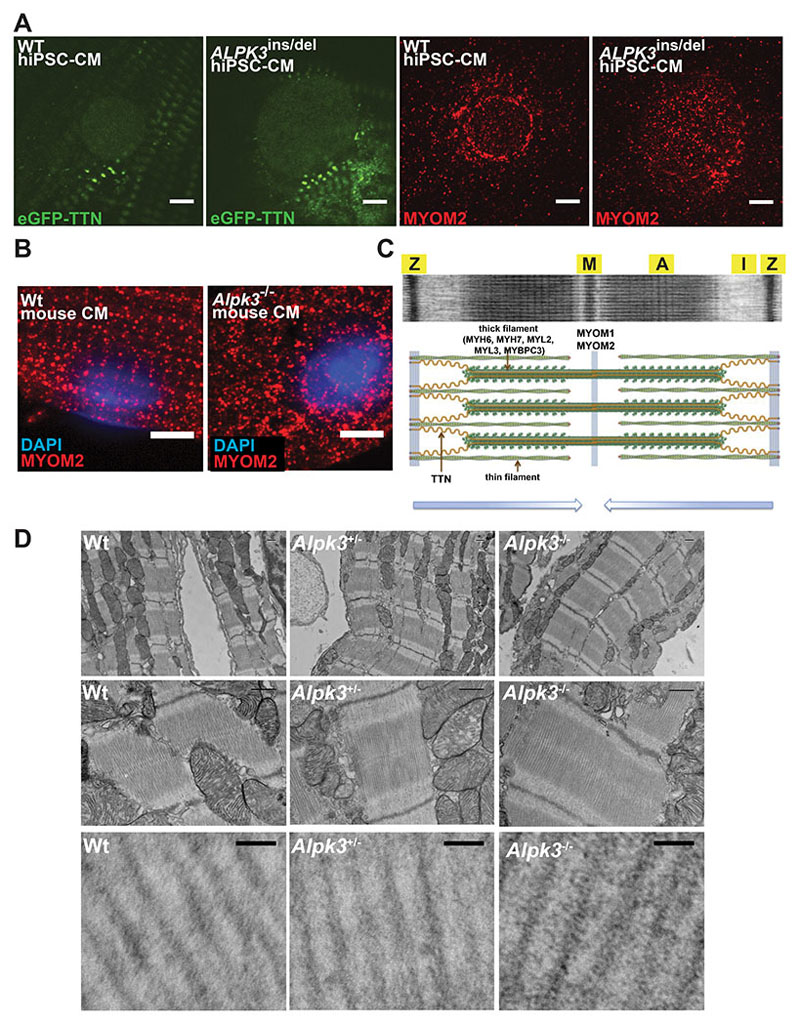
Confocal micrographs and electron microscopy demonstrate the effects of ALPK3 deficiency on the localization of myomesins and sarcomere structure in cardiomyocytes. **(A)** Confocal micrographs of WT and *ALPK3*^ins/del^ hiPSC-CMs expressing eGFP-TTN (green), probed with MYOM2 antibody (red). **Left panels –** eGFP-TTN expression in WT and *ALPK3*^ins/del^ nuclei is indistinguishable. **Right panels –** Prominent localization of myomesin-2 of the nuclear lamina in WT compared to diffuse staining across the nucleus in *ALPK3*^ins/del^ CMs. Scale bar, 5μm. **(B)** MYOM2 immunofluorescence is organized into striations at the M-band in Wt murine LV cardiomyocytes, but scattered without striations in *Alpk3*^-/-^ murine LV cardiomyocytes (isolated from mice age 7-9 weeks). Scale bar = 5μm. **(C)** Electron micrograph and schematic of sarcomere (modified from Garfinkel et al., *Heart Fail. Clin.* 2018 with permission). Z-discs (Z), M-band (M), A-band (A), I-band (I). (**D)** Electron micrographs demonstrate organized sarcomeres in Wt and *Alpk3*^+/-^ murine LV cardiomyocytes, while sarcomeres in *Alpk3*^-/-^ murine LV cardiomyocytes have less organized M-bands, loss of the central dark M-band stripe (see panel C) and increased electron density between thick filaments in the A-band region (lower panels). LV tissues isolated from mice age 4-8 weeks. A=A-band, M=M-band, scale bar (top two rows) = 500nm, (bottom row) = 50nm.

In hiPSC-CMs, antibodies to MYOM2 stained the nuclear envelope, a localization that has not been reported previously, hinting at unrecognized developmental roles for MYOM2 in immature cardiomyocytes. Relative to WT, MYOM2 staining of *ALPK3*^ins/del^ hiPSC-CMs was distributed more diffusely throughout the nucleus (Figure 5A). While Myom2 antibody staining of isolated murine LV cardiomyocytes (age 7-9 weeks) had poor signal-to-noise intensity, some striated staining at M-bands was observed in Wt cells, but no striations were discerned in *Alpk3*^-/-^ murine cardiomyocytes (Figure 5B).

The altered localization of myomesins in *Alpk3*^ins/del^ prompted a more detailed examination of *in vivo* sarcomere structures (Figure 5C). Electron microscopy of LV tissues (perfused with fixative in cardioplegic buffer to promote sarcomere relaxation) showed organized sarcomeres with discrete M-, A-, and I-bands in Wt and *Alpk3*^+/-^ mice (Figure 5D). In contrast, M-bands in *Alpk3*^-/-^ mice were less aligned and had enhanced electron density between the thick filaments in the peri M-band and A-band regions (Figure 5D), possibly due to mislocalized and accumulated M-band and thick filament proteins.

### ALPK3 co-localizes with myomesin proteins in the nucleus and the sarcomere in hiPSC-CMs

**Figure 6:**
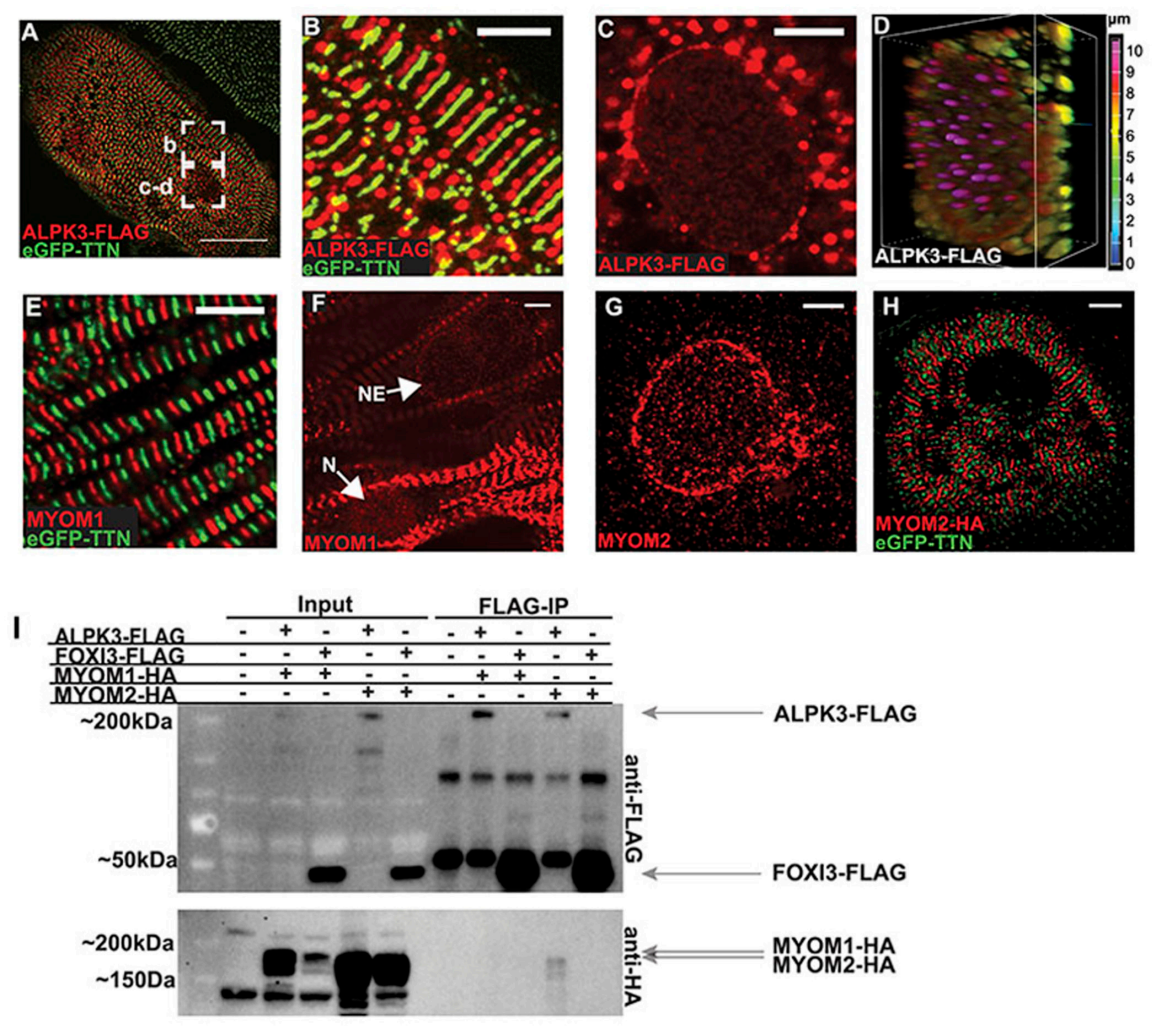
Colocalization and protein interactions of ALPK3 and myomesins. Confocal micrographs of hiPSC-CMs expressing eGFP-TTN (green) and ALPK3-FLAG (probed with anti-FLAG antibody; red). **(A)** ALPK3-FLAG introduced via transient plasmid transfection into hiPSC-CMs expressing eGFP-TTN. Scale bar = 25μm. **(B)** Magnified regions of panel A displays ALPK3-FLAG (red) localizing to sarcomere M-bands between Z-discs (green, eGFP-TTN). Scale bar = 5μm. **(C)** ALPK3-FLAG (red) localizes to nuclear envelope. Scale bar = 5μm. **(D)** Depth-encoded (Z-stack) maximum intensity projection of nucleus (panel C) showing ALPK3 localization at nuclear envelope. **(E)** MYOM1, stained with anti-myomesin 1 antibody (red) localizes to M-bands between Z-discs (green, eGFP-TTN). Scale bar = 5μm. **(F)** MYOM1 (red) is also found at the nucleus (N) and nuclear envelope (NE). Scale bar, 5μm. **(G)** MYOM2, stained with anti-myomesin 2 antibody (red) localizes to the nuclear envelope. Scale bar, 5μm. **(H)** hiPSC-CMs transfected with plasmid expressing MYOM2-HA (red) shows MYOM2 at M-bands between Z-discs (green, eGFP-TTN). Scale bar, 5μm. **(I)** Immunoprecipitation (IP) studies of ALPK3-FLAG, FOXI3-FLAG (negative control) and myomesins (MYOM1-HA, MYOM2-HA) in transfected HEK293T cells. Immunoprecipitation of ALPK3-FLAG results in pulldown of MYOM2-HA.

ALPK3 has a nuclear localization sequence and localizes primarily to the nucleus in transfected fibroblast-like COS cells,^39^ but its subcellular localization in cardiomyocytes was undetermined. As several commercial ALPK3 antibodies were non-specific (based on indistinguishable staining of *ALPK3*^ins/del^ and WT hiPSC-CMs), we produced an epitope tagged ALPK3 construct (ALPK3-FLAG) which was expressed in hiPSC-CMs via transient transfection. Using an anti-FLAG antibody (Figure 6A) we detected ALPK3 both at the M-band (Figure 6B) and the nuclear envelope (Figure 6C) in hiPSC-CMs. Depth-encoded projections of confocal z-slices confirmed that the majority of nuclear ALPK3 signal was at the nuclear envelope, as opposed to within the nucleus (Figure 6D).

Close examination of MYOM1 staining showed that in addition to its localization at the M-band (Figure 6E), some hiPSC-CMs also have MYOM1 at the nuclear envelope (Figure 6E) and within the nucleus (Figure 6F). While MYOM2 antibodies only stained the nuclear envelope in hiPSC-CMs (Figure 6G), the relative expression of MYOM2 in hiPSC-CMs and embryonic LV tissue is low (Figure S5D-E). We hypothesized that MYOM2 might undergo a post-natal shift to the M-band when its expression increases. Consistent with this model, increasing MYOM2 expression in hiPSC-CMs via transient transfection of an MYOM2-HA construct led to its prominent localization at the M-band (Figure 6H). Co-immunostaining confirmed that ALPK3-FLAG precisely colocalized with both MYOM1 and MYOM2-HA (Figure S8).

Ectopically expressed ALPK3-FLAG and MYOM2-HA were found to co-immunoprecipitate in HEK293T cells, indicating the presence of a direct interaction between these proteins. ALPK3-FLAG and MYOM1-HA did not coimmunoprecipitate in HEK293T cells but colocalized within cells (Figure S8), possibly because these proteins interact indirectly, or require additional cardiomyocyte-specific binding partners or factors which are not present in HEK293T cells.

Together, these analyses show that ALPK3, MYOM1, MYOM2 form a critical protein network in both the nucleus and sarcomere M-band at various stages in cardiomyocyte development and demonstrate that loss of *ALPK3* causes the profound mislocalization and accumulation of myomesin and thick filament proteins.

## Discussion

We report the biochemical and molecular functions of ALPK3 and provide insights into the pathogenesis of *ALPK3*-cardiomyopathy. Our investigations revealed that ALPK3 lacks catalytic activity, establishing it as the first pseudokinase of the α-kinase family. Employing multiple model systems including hiPSC-CMs, mice, and human patient tissues, we demonstrate that ALPK3 is essential for ensuring the proper localization and expression of proteins including myomesins in both the nucleus and sarcomere, the absence of which compromises cardiomyocyte structure and function.

ALPK3 had not been previously identified as a pseudokinase, although distinguishing these from active kinases using bioinformatic approaches remains a difficult and imperfect science. By comparing the α-kinase domains in ALPK3 with other human α-kinases and their orthologs we found that there were four evolutionarily conserved residues (G1725, K1729, Q1769, N1797) which were all variant in ALPK3. Our phosphoproteomic studies of *ALPK3*^dIC/dIC^ hiPSC-CMs, which altered the conformation of the kinase domain, *ALPK3*^sIC/sIC^ which targeted the predicted aspartate residue critical for catalysis, and massive overexpression of ALPK3 in HEK293T cells indicated that none of these perturbations significantly affected phosphorylation across the proteome. Given the perfect conservation of four residues within all other α-kinase family members except for ALPK3, we infer that the variation of these residues during evolution may have inactivated ALPK3 catalytic functions and enabled the kinase domain to assume other, noncatalytic functions.

By exploring these functions in both human and mouse cardiomyocytes, we identified a novel subcellular localization of ALPK3 at the sarcomere M-band and the nuclear envelope and revealed that ALPK3 is critical for ensuring the proper localization of myomesin proteins at these regions. The dysregulation and mislocalization of myomesin proteins that occurred with *ALPK3*^dIC/dIC^ and *ALPK3*^ins/del^ variants indicates that the α-kinase domain may function in recruiting myomesin proteins at these structures, as supported by coimmunoprecipitation experiments which showed a direct interaction between ALPK3 and MYOM2. Myomesins are spring-like proteins containing multiple α-helices that undergo rapid unfolding and refolding under axial forces, minimizing M-band force imbalances generated by opposing myosins during sarcomere contraction.^33,34,40^ Given these functions, we propose that *ALPK3* variants which cause mislocalization of myomesins may compromise force buffering and lead to impaired structural integrity of the sarcomere, as was seen by examination of *Alpk3*^-/-^ murine LV tissues by electron microscopy. Rare loss-of-function and deleterious missense variants in *MYOM1* and *MYOM2* are associated with human cardiomyopathies, and knockout of *MYOM1* in hiPSC-CMs and *MYOM2* in *Drosophila* have deleterious effects on sarcomere structure and contractility, further indicating the functional importance of these proteins in cardiomyocytes.^41–43^

**Figure 7:**
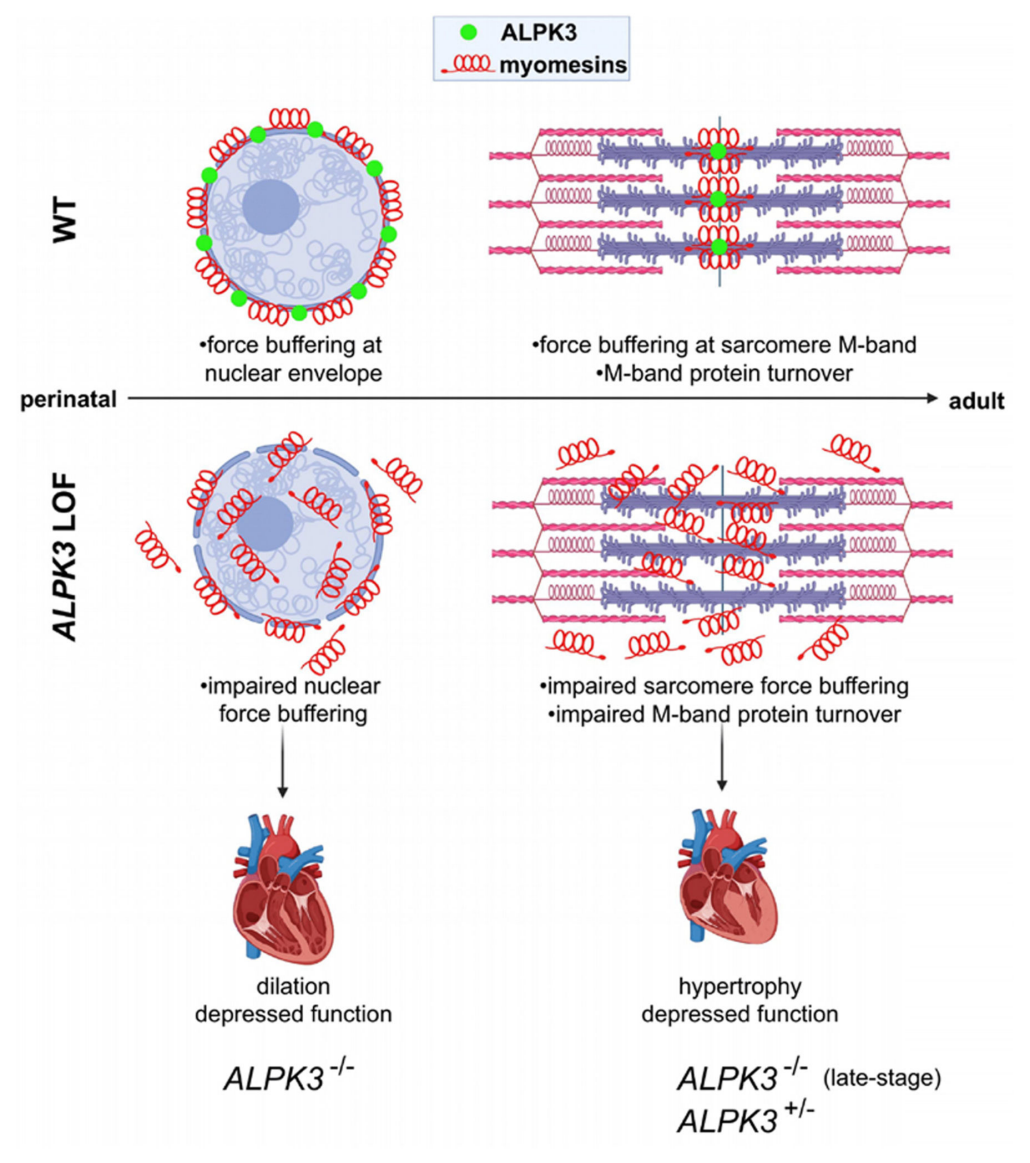
Model for ALPK3 function in cardiomyocytes. **(Top)** In WT cardiomyocytes, ALPK3 (green) is found in both the nuclear envelope and the sarcomere M-band together with myomesin proteins (red). Myomesins are spring-like proteins with multiple distensible α-helical domains which can buffer mechanical forces. In the perinatal period, myomesins are found in the nuclear envelope and later in development, they are found in the sarcomere. **(Bottom)** *ALPK3* loss-of-function (LOF) variants cause myomesin proteins to mislocalize and accumulate, compromising nuclear and sarcomeric force buffering leading to cardiomyocyte dysfunction, ventricular dilatation, and rapid heart failure. Emergence of hypertrophy in neonatal survivors and adults with *ALPK3* heterozygous variants may be caused by the dysregulation of additional M-band proteins including depressed MuRF1 and increased CAPN3 levels, which participate in sarcomere protein turnover.

While the importance of myomesin proteins in the sarcomere is well-established, the nuclear roles of these proteins are not well understood.^44^ Although MYOM1 was first recognized as a nuclear protein in studies of newborn rat cardiomyocytes, we further demonstrated that MYOM1, MYOM2, and ALPK3 are all present in the nuclear envelope of hiPSC-CMs, suggesting an early developmental function of this protein network. The importance of the nuclear envelope for cardiomyocyte function is demonstrated by loss-of-function variants in lamin A/C (*LMNA*), which compromise nuclear envelope integrity and lead to dilated cardiomyopathy.^45,46^ We speculate that the insertion of myomesins in the nuclear envelope in developing cardiomyocytes may also confer force buffering functions, thereby protecting the nucleus in the neonatal period when cardiomyocytes must divide and replicate while maintaining cardiac contraction. Thus, myomesin mislocalization in both the nucleus and sarcomere may ultimately impair cardiomyocyte structure and function, culminating in depressed contractility and ventricular dilatation (Figure 7). Future studies to specifically ablate the nuclear or sarcomeric populations of ALPK3 and myomesins at various developmental timepoints may help to discern the precise roles of these proteins at either structure.

We suspect that the hypertrophy caused by *ALPK3*-cardiomyopathy in surviving newborns with recessive pathogenic variants and adults with heterozygous pathogenic variants may be related to additional roles of ALPK3 in directly or indirectly regulating sarcomere protein turnover at the M-band. Another M-band pseudokinase domain is encoded by the C-terminus of titin (TTN) which recruits several proteins important in sarcomere protein turnover including the autophagy cargo receptor (encoded by *NBR1*), p62 (encoded by *SQSTM*), and MuRF1 (encoded by *TRIM63*) complex, which degrades thick filament proteins,^37^ and the cysteine protease calpain-3 (encoded by CAPN3) which cleaves and releases myofibrillar proteins for ubiquitin-proteasome-dependent degradation.^47–49^ We found that MuRF1 and CAPN3 were both differentially expressed in *ALPK3*^dIC/dIC^ and *ALPK3*^ins/del^ hiPSC-CMs, suggesting that loss of the ALPK3 kinase domain may also dysregulate this protein turnover machinery. The dysregulation of MuRF1 is particularly remarkable, as this E3-ubiquitin ligase targets contractile proteins. Like ALPK3, MURF1 expression is developmentally regulated, increasing late in gestation to achieve a maximal level that is maintained throughout life,^50^ and can be found in both the nucleus and M-band.^51^ Augmentation of MuRF1 expression promotes muscle atrophy^37^, while reduction in MuRF1 expression caused by rare recessive human pathogenic variants in *TRIM63* promotes hypertrophic remodeling.^52^ We speculate that the reduced levels of MuRF1 protein (despite normal transcripts) in *ALPK3*^ins/del^ and *ALPK3*^dIC/dIC^ hiPSC-CMs could reflect its heightened consumption, which may contribute to the accumulation of M-band and thick filament proteins that was observed in *ALPK3* mutant hiPSC-CMs. This could explain the eventual emergence of hypertrophy in surviving neonates who initially present with cardiac dilation, and in adults with heterozygous *ALPK3* variants^6^ (Figure 7).

In summary, our studies identify ALPK3 as an α-pseudokinase which regulates the expression and localization of critical proteins including myomesins in both the sarcomere M-band and the nuclear envelope of cardiomyocytes. Further studies to characterize the ALPK3-MYOM1-MYOM2 protein network across developmental stages will provide further insights into the roles of these proteins in the establishment and maintenance of cardiomyocyte structure and function.

## Supplementary Material

Supplemental Material 6

Supplemental Review Material File_1

Supplemental Review Material File_2

Supplemental Review Material File_3

Supplemental Review Material File_4

## Figures and Tables

**Table 1:** Isogenic hiPSC models with biallelic *ALPK3* mutations^

*ALPK3* mutant hiPSC line	*ALPK3* cDNA Δ	Variant Class	ALPK3 protein level
*ALPK3* ^ins/del^	c.2442_2443insA c.2428_2458del31	Frameshift Frameshift	<5% WT
*ALPK3* ^dIC/dIC^	c.5330_5331GG>AA (homozygous) c.5351A>C (homozygous)	Missense, G1777E Missense, D1784A	~2X WT
*ALPK3* ^del/del^	c.5306_5325del20 c.5310_5320del11	Frameshift Frameshift	~39% WT
*ALPK3* ^sIC/sIC^	c.5321A>C (homozygous)	Missense, D1774A	~WT

^ See Figure S1 for sequence validation and Figure S2F,H for ALPK3 protein level quantitation.

## Clinical Perspective

### What is new?

- Damaging variants in *ALPK3,* encoding an abundant muscle-specific protein, cause both neonatal and adult-onset cardiomyopathies and lead to both ventricular dilatation and hypertrophy.
- Although ALPK3 contains an α-kinase domain, we show that it lacks catalytic activity and is a pseudokinase.
- ALPK3 localizes to both the nuclear envelope of cardiomyocytes and the M-band of the sarcomere where it regulates the expression and localization of myomesins (MYOM1, MYOM2) and additional M-band proteins important for sarcomere protein turnover.

### What are the clinical implications?

- *ALPK3*-cardiomyopathy may cause impaired contractility and ventricular dilatation due to mislocalization and dysregulation of myomesin proteins which are critical for force buffering in cardiomyocytes.
- *ALPK3*-cardiomyopathy may cause hypertrophy due to dysregulation of key M-band proteins which are important for sarcomere protein turnover.
- Therapeutic strategies to restore cardiomyocyte force buffering functions and sarcomere protein turnover may ameliorate disease phenotypes in patients with *ALPK3*-cardiomyopathy.
