# Supplementary material for: Pathogenesis of Cardiomyopathy Caused by Variants in ALPK3, an Essential Pseudokinase in the Cardiomyocyte Nucleus and Sarcomere
Source: Circulation. 2022 Nov 2;146(22):1674–93. doi: 10.1161/CIRCULATIONAHA.122.059688 (PMC9698156; doi:10.1161/CIRCULATIONAHA.122.059688)
Supplement: Supplementary file 1 [file cir-146-1674-s001.pdf]

SUPPLEMENTAL MATERIAL for:

Pathogenesis of cardiomyopathy caused by variants in *ALPK3*, an essential pseudokinase in the cardiomyocyte nucleus and sarcomere

Radhika Agarwal MD, PhD, Hiroko Wakimoto MD, PhD, Joao A. Paulo PhD, Qi Zhang MD, Daniel Reichart MD, Christopher Toepfer PhD, Arun Sharma PhD, Angela C. Tai BA, Mingyue Lun MD, PhD, Joshua Gorham BA, Steven R. DePalma PhD, Steven P. Gygi PhD†, J. G. Seidman PhD† and Christine E. Seidman MD†

† Denotes equal contribution.

Supplemental Tables

Table S1. Predicted functions of α-kinase domain residues from TRPM7[10,19] and their homologous residues in ALPK3. Residues (red) are conserved in all human α-kinases. Residues (green) are conserved in all α-kinases except ALPK3 (ALPK3-specific nonconserved residues). Asterisk (*****) indicates conserved residues that were targeted for mutagenesis by CRISPR/Cas9. See also Figure 1b-c and Figure S1.

Homologous ALPK3 residue	TRMP7 residue	Function TRPM7
G1621	G1621	Binds γP of ATP
R1624	R1624	Binds γP of ATP
Q1677	Q1676	?
L1708	L1707	?
E1722	E1720	Bridge to K1648, ATP
K1727	G1725	?
S1731	K1729	May bridge to γP of ATP
H1760	H1753	Chelates zinc
V1772	V1765	?
D1774*	D1767	**Catalytic aspartate**
A1776	Q1769	Orients ATP, may chelate Mg^{2+}
G1777*	G1770	**?**
T1783	T1776	Hydrophobic pocket for ATP
D1784*	D1777	**Orients ATP, may chelate Mg^{2+}**
S1800	N1797	May participate in substrate binding
F1809	F1806	Hydrophobic packing in C-lobe
C1815	C1812	Chelates zinc
N1816	N1813	Binds C1816
C1819	C1816	Chelates zinc
L1824	L1821	Hydrophobic packing in C-lobe

Table S2. Genotypes and clinical characteristics of human left ventricular tissues from *ALPK3*-cardiomyopathy patients (PT1-PT2) and controls (C1-C5).

**Tissue**	***ALPK3* Genotype**	**ALPK3 Predicted Protein Δ**	**Age**	**Sex**	**Cause of death**	**Other**
C1	Control	Control	54	F	head trauma	-
C2	Control	Control	43	F	suicide	-
C3	Control	Control	52	M	suicide	-
C4	Control	Control	53	M	ruptured cerebral aneurysm	-
C5	Control	Control	48	F	stroke	-
PT1	c.1018 C>T c.2434 G>A	p.Q340X p.V812M	4	M	-	Tissue acquired at time of transplant
PT2	c.1018 C>T c.4335 A>G	p.Q340X p.K1445RfsTer29	28	F	-	Tissue acquired at time of transplant

Table S3. Transcript levels of myomesin and myosin heavy chain in WT and *ALPK3* mutant hiPSC-CMs and mice as measured by bulk RNAseq. Data are mean±SEM.

	**WT hiPSC-CMs (n=13)**	***ALPK3***$^{-/-}$ **hiPSC-CMs (n=3)**	**p-value**	**WT mouse LV (n=3)**	***Alpk3***$^{-/-}$ **mouse LV (n=3)**	**p-value**
MYOM1	102±16	153±30	0.16	241±9	333±43	0.06
MYOM2	1.8±0.9	3.3±0.5	0.46	172±4	216±3	**4.2×10^{-4}**
MYH6	309±79	1269.1±78	**4.5×10^{-5}**	6790±45	5796±237	**7.3×10^{-3}**
MYH7	1800±214	1835.5±353	0.94	163±4	201±27	0.17

Table S4. Transcript levels of myomesin and myosin heavy chain in cardiomyocytes from *ALPK3*-cardiomyopathy human patient left ventricular tissues versus controls (see also Table S2), as measured by single nuclear RNAseq. Data are mean RNA expression.

	**Controls**	***ALPK3* patient tissue**	**p-value**
MYOM1	22.0±7.1	31.4±4.5	0.12
MYOM2	10.4±2.3	14.5±9.2	0.64
MYH6	4.0±2.2	2.1±0.8	0.11
MYH7	11.0±1.8	20.4±12.9	0.49

Supplemental Figures

Figure S1.

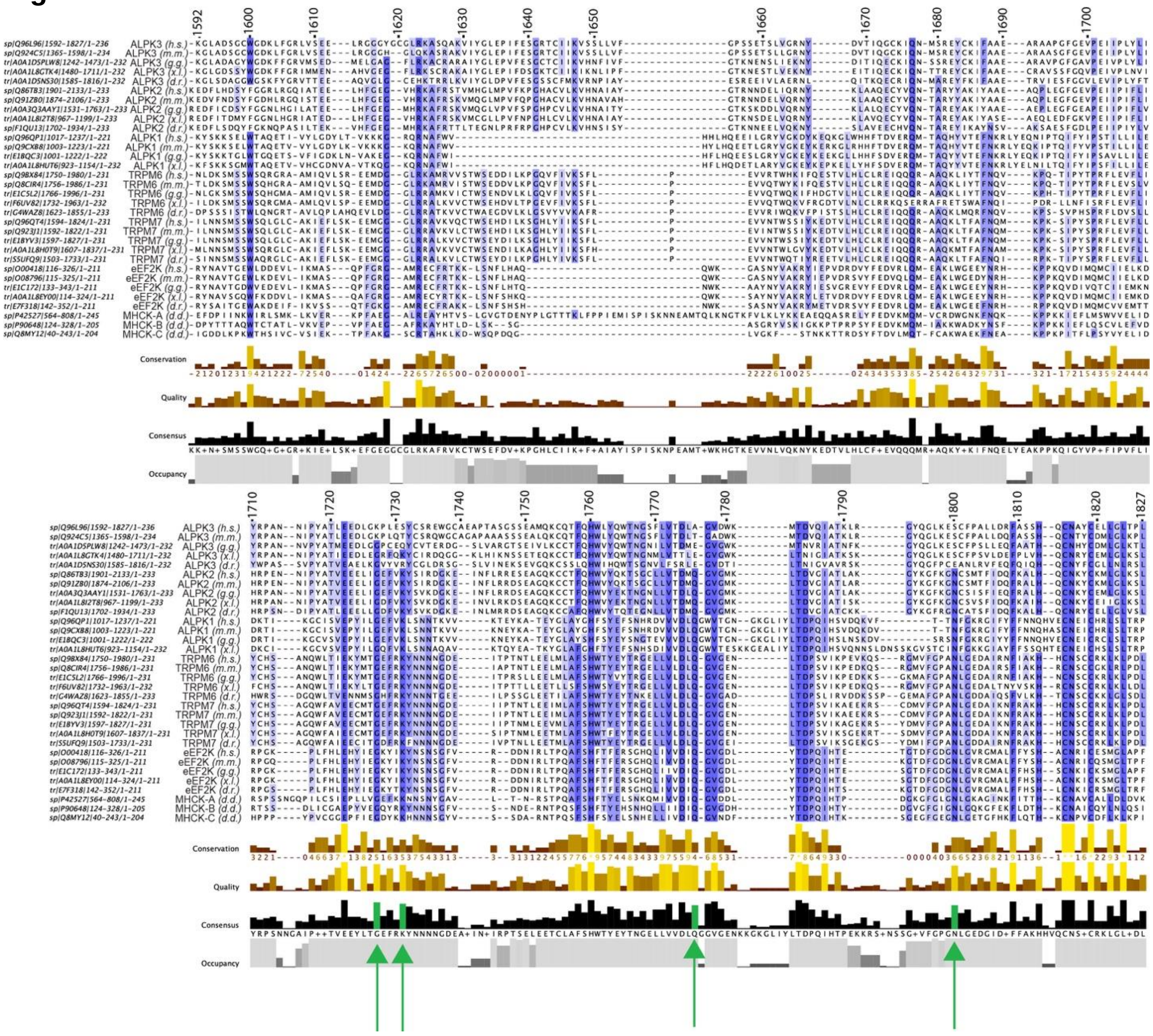

Figure S1. Multiple sequence alignment of α-kinase domains from human α-kinases and their orthologs. Clustal Omega[16] alignment of all human α-kinase domains (ALPK3, ALPK2, ALPK1, TRPM6, TRPM7, eEF2K) and their orthologs in (*m.m. = mus musculus [mouse], g.g. = gallus gallus [chicken], x.l. = xenopus laevis [frog] d.r. = danio rerio [zebrafish]*, and *dictyostelium discoideum* (*d.d*, slime mold). Leftmost column indicates Uniprot accession IDs and protein names. Residue positions are numbered according to the relative position in the ALPK3 human protein sequence. Green arrows highlight residues that are conserved in all α-kinase domains <u>except</u> for ALPK3 (ALPK3-specific nonconserved residues) (see also Figure 1b-c and Table S1).

Figure S2.

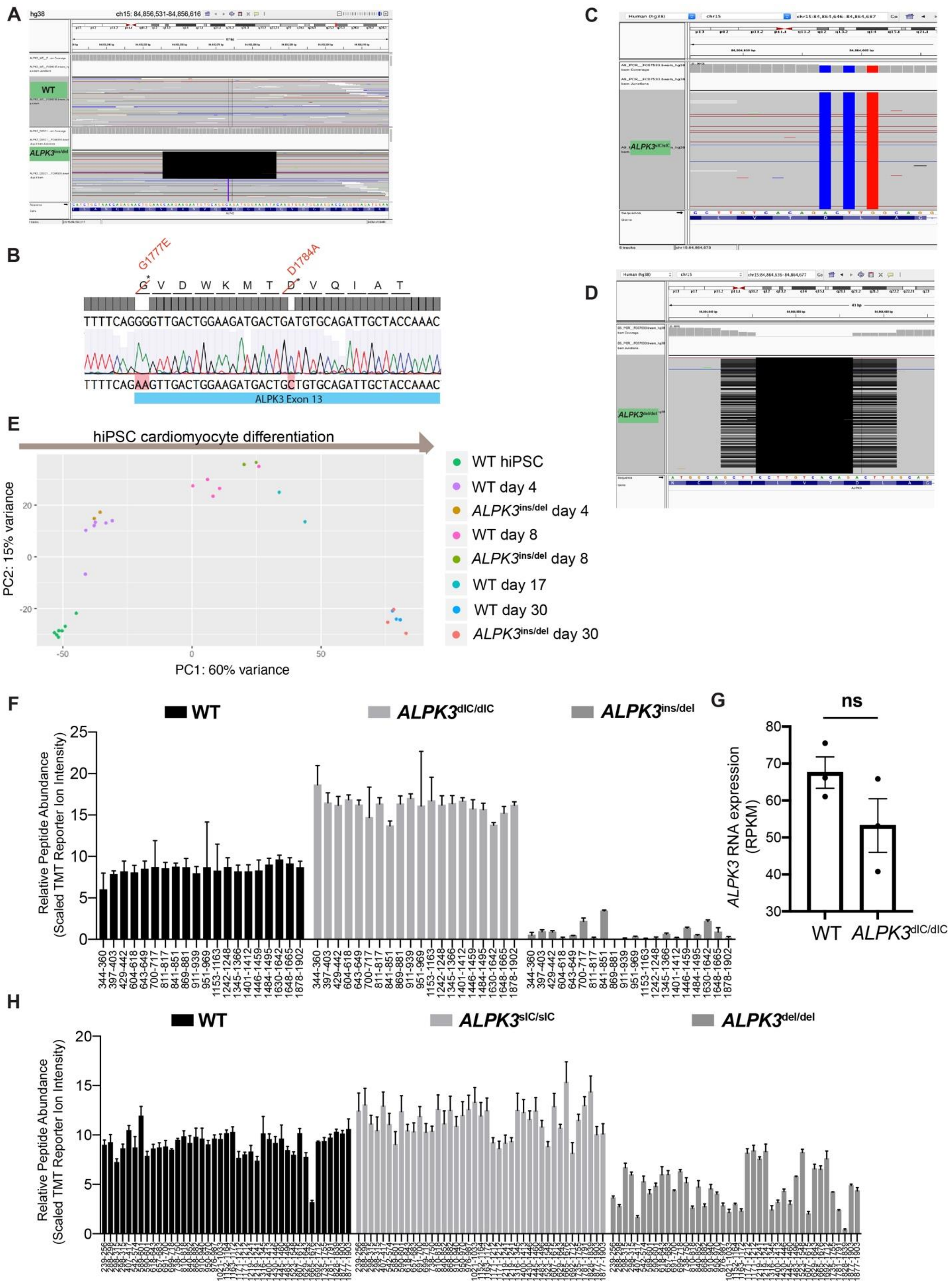
A
hg38
ch15: 84,856,531-84,856,616
WT
ALPK3ins/del
[redacted]
C
ALPK3dIC/dIC
D
ALPK3del/del
B
G1777E
D1784A
G V D W K M T D V Q I A T
TTTTCAGGGGTTGACTGGAAGATGACTGATGTGCAGATTGCTACCAAAC
TTTTCAGAAGTTGACTGGAAGATGACTGCTGTGCAGATTGCTACCAAAC
ALPK3 Exon 13
E
hiPSC cardiomyocyte differentiation
PC2: 15% variance
PC1: 60% variance
WT hiPSC
WT day 4
ALPK3ins/del day 4
WT day 8
ALPK3ins/del day 8
WT day 17
WT day 30
ALPK3ins/del day 30
F
WT
ALPK3dIC/dIC
ALPK3ins/del
Relative Peptide Abundance
(Scaled TMT Reporter Ion Intensity)
G
ns
ALPK3 RNA expression
(RPKM)
WT
ALPK3dIC/dIC
H
WT
ALPK3sIC/sIC
ALPK3del/del
Relative Peptide Abundance
(Scaled TMT Reporter Ion Intensity)

Figure S2. Sequence validation and characterization of *ALPK3* hiPSC mutants
Representative IGV traces from MiSeq of **(A)** *ALPK3*$^{ins/del}$ hiPSCs **(B)** *ALPK3*$^{dIC/dIC}$ hiPSCs **(C)** *ALPK3*$^{del/del}$ hiPSCs **(D)** *ALPK3*$^{sIC/sIC}$ hiPSCs (see also Table 1 for genotypes) **(E)** Principal component analysis of RNAseq from WT and *ALPK3*$^{ins/del}$ hiPSCs at various stages of cardiomyocyte differentiation (day 4 = cardiac mesoderm, day 8 = early cardiac progenitor, day 17 = early cardiomyocyte, day 30 = cardiomyocyte). **(F)** ALPK3 protein expression in WT (n=4), *ALPK3*$^{dIC/dIC}$ (n=4) and *ALPK3*$^{ins/del}$ (n=3) hiPSC-CMs. Data are mean±SEM. For f & h x-axis indicates the peptide position within the ALPK3 protein sequence (length = 1,907 amino acids). **(G)** *ALPK3* RNA levels in WT (n=3) and *ALPK3*$^{dIC/dIC}$ hiPSC-CMs (n=3), as measured by RNAseq. **(H)** ALPK3 protein expression in WT (n=4), *ALPK3*$^{sIC/sIC}$ (n=4) and *ALPK3*$^{del/del}$ (n=4) hiPSC-CMs. Data are mean±SEM.

Figure S3.

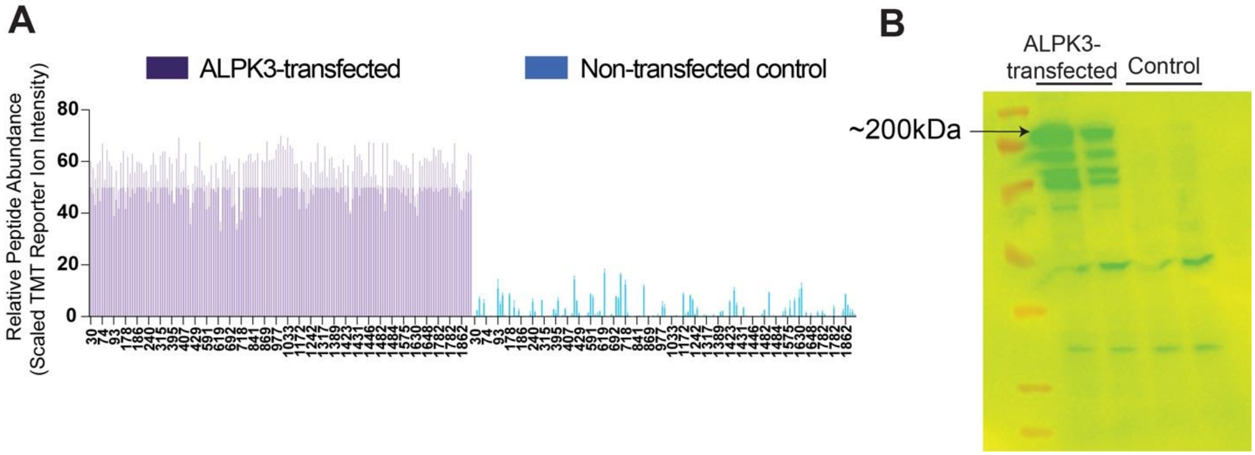

Figure S3. ALPK3 expression in transiently transfected and control HEK293T cells. (a) Relative ALPK3 peptide expression in ALPK3-transfected (n=2) versus control (n=2) HEK293T cells, quantified by mass-spectrometry. A total of 33 peptides mapped uniquely to ALPK3; the start position of each of these peptides in the ALPK3 protein is indicated on the x-axis. Data depicted as mean±SEM. **(b)** Western blot of protein lysates harvested from ALPK3-transfected and control HEK293T cells. Blot was probed using an anti-FLAG antibody to detect ALPK3-FLAG protein. ALPK3 molecular weight = 201kDa.

Figure S4.

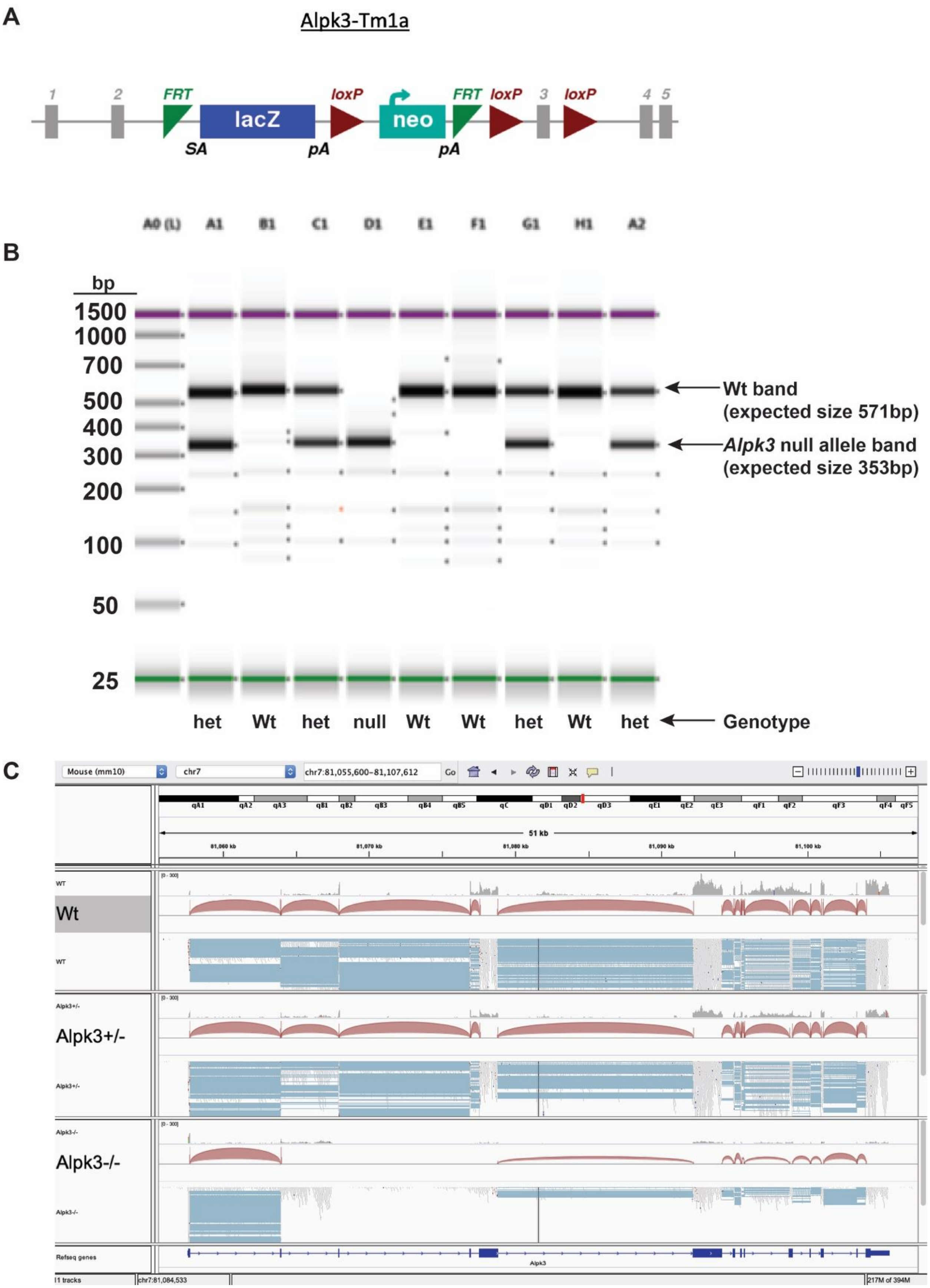

A
Alpk3-Tm1a
1
2
FRT
lacZ
loxP
neo
FRT
loxP
3
loxP
4
5
SA
pA
pA
B
A0 (L)
A1
B1
C1
D1
E1
F1
G1
H1
A2
bp
1500
1000
700
500
400
300
200
100
50
25
Wt band
(expected size 571bp)
Alpk3 null allele band
(expected size 353bp)
het
Wt
het
null
Wt
Wt
het
Wt
het
Genotype
C
Mouse (mm10)
chr7
chr7:81,055,600-81,107,612
Go
51 kb
Wt
Alpk3+/-
Alpk3-/-
Refseq genes
Alpk3
11 tracks
chr7:81,084,533
217M of 394M

Figure S4. Genotyping validation of *Alpk3* mouse model. **(A)** *Alpk3*$^{+/-}$ mice were created by MRC Harwell by crossing mice containing the Tm1a allele (shown above) with mice expressing the Cre recombinase. **(B)** Representative image of PCR amplicons run on Agilent TapeStation (Methods) confirming mouse genotypes. Detected bands for *Wt* and *Alpk3*-null alleles match expected sizes. **(C)** Representative integrated genome viewer (IGV) trace confirms absence of *Alpk3* transcripts in exon 3 region of *Alpk3*$^{-/-}$ mice (RNAseq of mouse left ventricular tissues at postnatal day 8).

Figure S5.

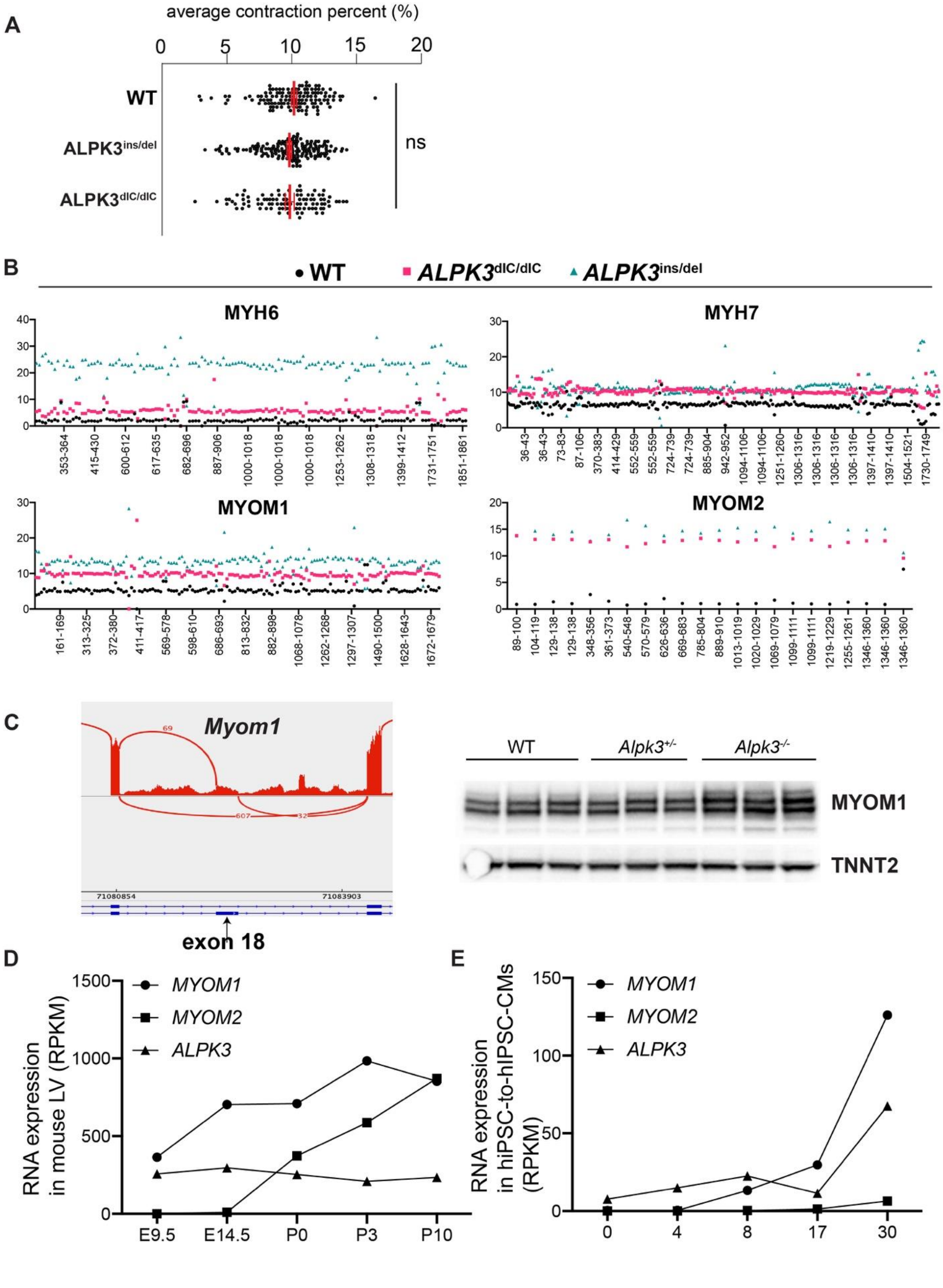
A
average contraction percent (%)
0
5
10
15
20
WT
ALPK3ins/del
ALPK3dIC/dIC
ns
B
WT
ALPK3dIC/dIC
ALPK3ins/del
MYH6
MYH7
MYOM1
MYOM2
C
Myom1
69
607
32
71080854
71083903
exon 18
WT
Alpk3+/-
Alpk3-/-
MYOM1
TNNT2
D
RNA expression in mouse LV (RPKM)
MYOM1
MYOM2
ALPK3
E9.5
E14.5
P0
P3
P10
E
RNA expression in hiPSC-to-hiPSC-CMs (RPKM)
0
4
8
17
30

Figure S5. (A) Sarcomere contractile function in WT and *ALPK3* mutant hiPSC-CMs, assessed with SarcTrack. Each point represents the averaged sarcomere contractility from one video (v), containing (n) number of sarcomeres from (d) independent differentiations (WT: n=43,761, v=94, d=2; *ALPK3*$^{-/-}$: n=47,977, v=95, d=2; *ALPK3*$^{dIC/dIC}$: n=46,720, v=95, d=2). Data are mean±SEM. **(B)** Expression of myosin heavy chain and myomesin proteins in WT and *ALPK3* mutant hiPSC-CMs. Only nonredundant peptides that mapped uniquely to each protein are graphed. x-axis: peptide position in protein sequence. y-axis: relative peptide abundance (scaled TMT reporter ion intensity). (Data shown are means, WT n= 4; *ALPK3*$^{dIC/dIC}$ n=4; *ALPK3*$^{-/-}$ n=3) **(C)** IGV sashimi plot of *MYOM1* splice isoforms. The shorter splice isoform results in the exclusion of *MYOM1* exon 18. Western blot of MYOM1 expression in left ventricular tissue of P8 mice. Replicates represent protein lysates from different mice. Loading control: TNNT2. **(D)** RNA levels of MYOM1, MYOM2, and ALPK3 transcripts through mouse development. **(E)** RNA levels of MYOM1, MYOM2, and ALPK3 transcripts through hiPSC-CM differentiation.

Figure S6.

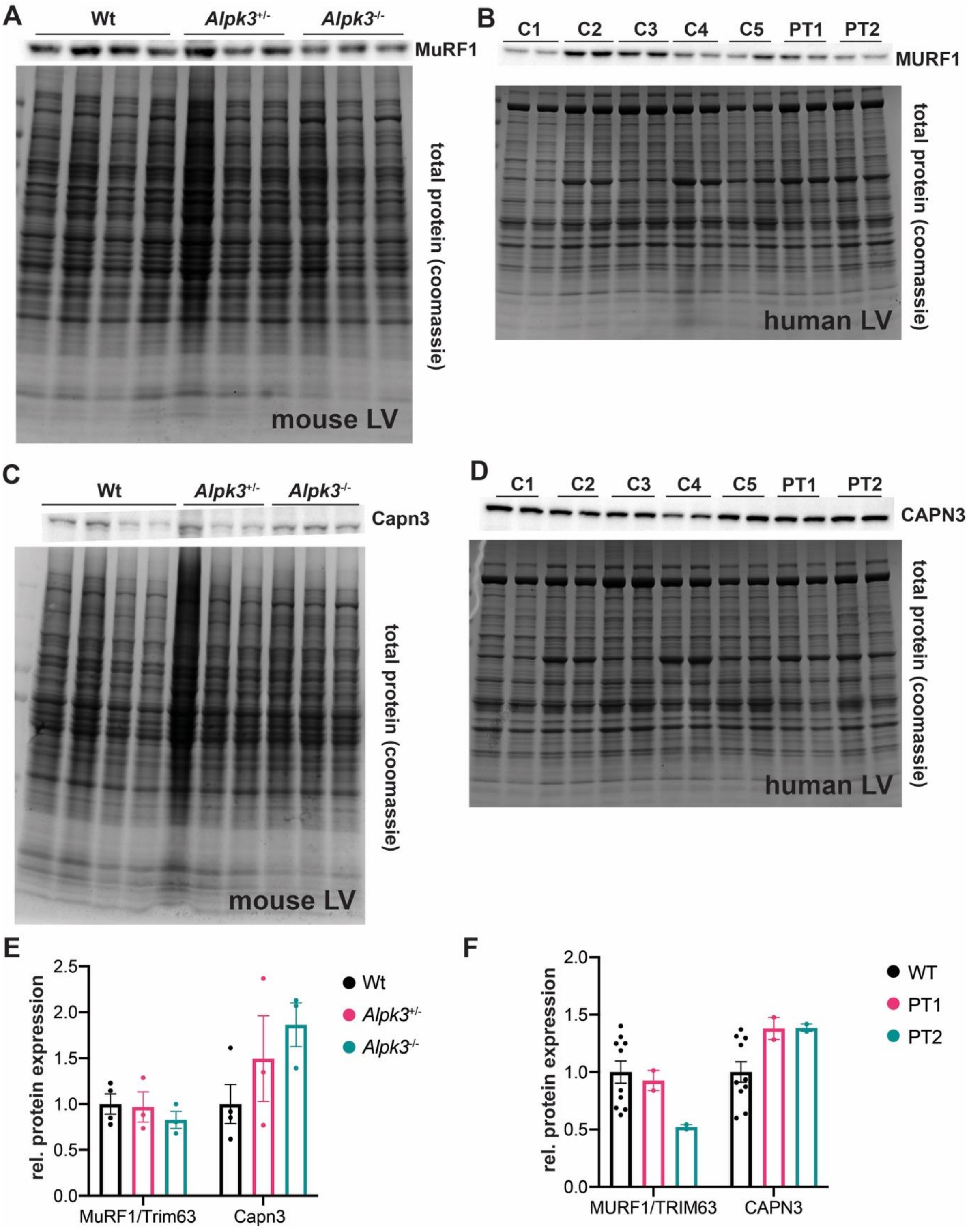

Figure S6. Western Blots of MURF1 **(A-B)** and CAPN3 (**C**-**D**) expression in WT and *ALPK3* mutant mouse LV tissues and human LV patient tissues. MURF1 or CAPN3

expression was normalized to total protein as detected by Coomassie stained protein gel and normalized to WT, quantified in **(E-F)**.

Figure S7.

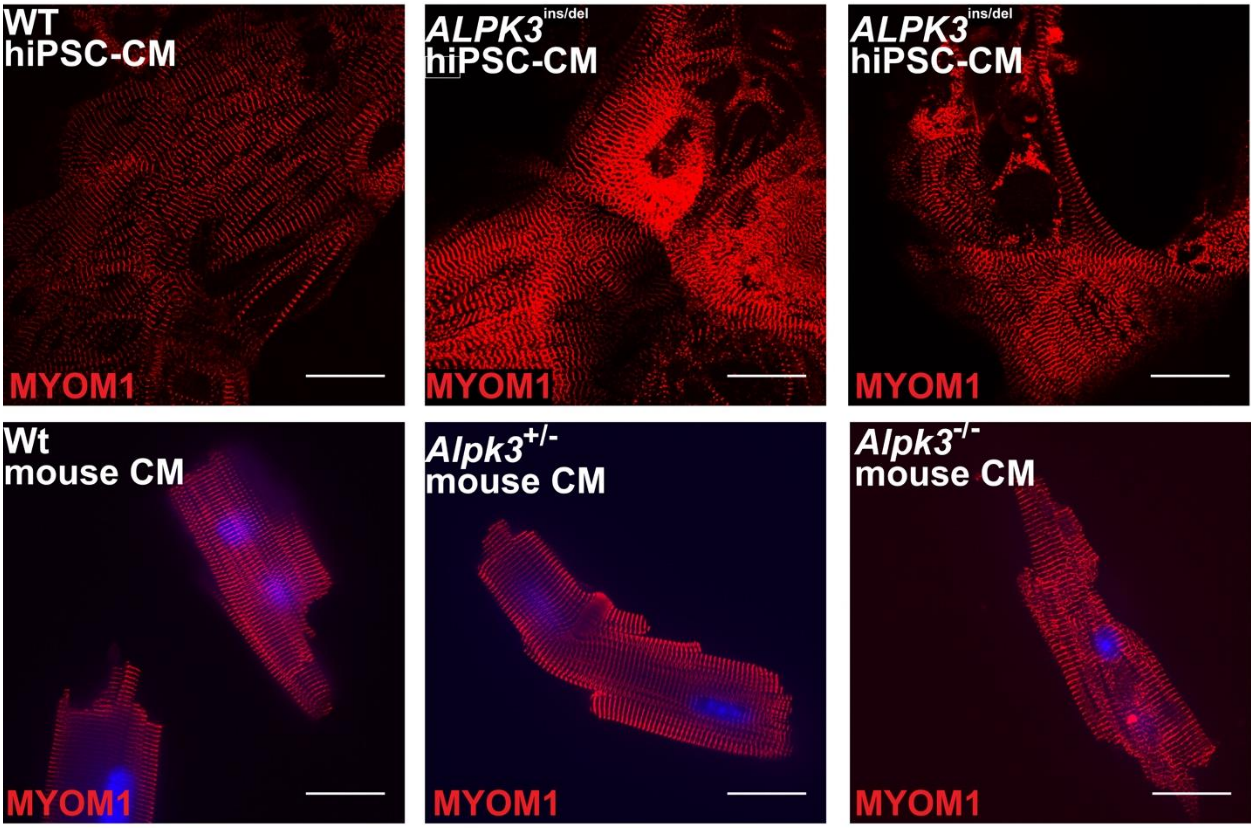

Figure S7. Complete confocal micrographs of WT, *ALPK3*$^{ins/del}$ hiPSC-CMs and murine cardiomyocytes stained with MYOM1 antibody (red). (Top Row) MYOM1 in hiPSC-CMs. **(Bottom row)** Myom1 in *Alpk3*$^{-/-}$ mouse cardiomyocytes (left ventricular cardiomyocytes isolated from mice age 7-9 weeks). Scale bar = 25μm. See also Figure 4.

Figure S8.

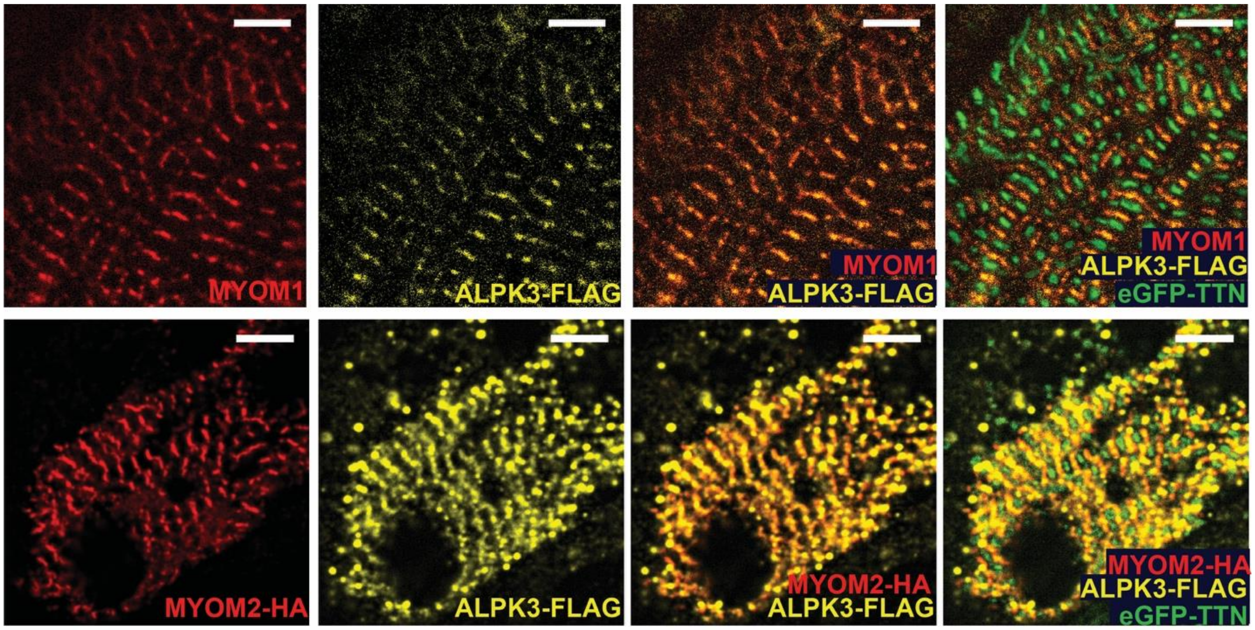

Figure S8. Colocalization of ALPK3-FLAG, MYOM1, and MYOM2-HA in hiPSC-CMs. Confocal micrographs of WT hiPSC-CM transfected with ALPK3-FLAG (top row) or ALPK3-FLAG and MYOM2-HA (bottom row). Scale bar = 5μm.

Supplemental Data Sets

Supplemental Data S1. ***Tab 1**.* RNAseq of WT and *ALPK3*$^{ins/del}$ hiPSCs through cardiomyocyte differentiation. Data represent reads per kilobase of transcript per million mapped reads (RPKM). ***Tab 2***. RNAseq (RPKM) of select cardiomyocyte differentiation markers in WT and *ALPK3*$^{ins/del}$ hiPSC-CMs at day 30.

Supplemental Data S2. ***Tab 1.*** Phosphopeptides detected by tandem-mass-tag mass spectrometry in day 30 WT, *ALPK3*$^{ins/del}$ and *ALPK3*$^{dIC/dIC}$ hiPSC-CMs. ***Tab 2.*** Set of 3 differentially phosphorylated sites in *ALPK3*$^{dIC/dIC}$ hiPSC-CMs. ***Tab 3.*** Set of 225 differentially phosphorylated sites in *ALPK3*$^{ins/del}$ hiPSC-CMs. ***Tab 4.*** Phosphopeptides detected by tandem-mass-tag mass spectrometry in day 30 WT, *ALPK3*$^{del/del}$ and *ALPK3*$^{sIC/sIC}$ hiPSC-CMs. ***Tab 5.*** Set of 19 differentially phosphorylated sites in *ALPK3*$^{sIC/sIC}$ hiPSC-CMs. ***Tab 6.*** Set of 15 differentially phosphorylated sites in *ALPK3*$^{del/del}$ hiPSC-CMs. ***Tab 7.*** Phosphopeptides detected by tandem-mass-tag mass spectrometry in mouse Wt, *Alpk3*$^{+/-}$ and *Alpk3*$^{-/-}$ left ventricular tissues at postnatal day 8. ***Tab 8.*** Set of 38 differentially phosphorylated sites in *Alpk3*$^{-/-}$ mouse LV tissues. There were no differentially phosphorylates sites in *Alpk3*$^{+/-}$ LV tissues. For all sheets: Blue columns represent phosphopeptide abundance, yellow columns represent protein abundance, orange columns represent phosphopeptide abundance normalized to protein abundance. Differential phosphorylation criteria: $\log_2$fold-change $\geq |0.67|$, unadjusted p-value < 0.01.

Supplemental Data S3. ***Tab 1.*** Proteins detected by tandem-mass-tag mass spectrometry in day 30 WT, *ALPK3*$^{ins/del}$ and *ALPK3*$^{dIC/dIC}$ hiPSC-CMs. ***Tab 2.*** Set of 27 differentially expressed (DE) proteins in *ALPK3*$^{dIC/dIC}$ hiPSC-CMs. ***Tab 3.*** Set of 264 DE proteins in *ALPK3*$^{ins/del}$ hiPSC-CMs. ***Tab 4.*** Proteins detected by tandem-mass-tag mass spectrometry in day 30 WT, *ALPK3*$^{sIC/sIC}$ and *ALPK3*$^{del/del}$ hiPSC-CMs. ***Tab 5.*** Set of 24 DE proteins in *ALPK3*$^{sIC/sIC}$ hiPSC-CMs. ***Tab 6.*** Set of 12 DE proteins in *ALPK3*$^{del/del}$ hiPSC-CMs. DE criteria: $\log_2$fold-change $\geq |0.67|$, unadjusted p-value < 0.01. ***Tab 7.*** Proteins detected by tandem-mass-tag mass spectrometry in Wt, *Alpk3*$^{+/-}$ and *Alpk3*$^{-/-}$ murine left ventricular tissues at postnatal day 8. ***Tab 8.*** Set of 38 differentially expressed (DE) proteins in *Alpk3*$^{-/-}$ murine LV tissues versus WT. DE criteria: $\log_2$fold-change $\geq |0.67|$, unadjusted p-value < 0.01.

Supplemental Data S4. ***Tab 1***. Phosphopeptides detected by tandem-mass-tag mass spectrometry in transiently-transfected ALPK3-FLAG HEK293T cells versus untransfected controls. ***Tab 2.*** Proteins detected by tandem-mass-tag mass spectrometry in transiently transfected ALPK3-FLAG HEK293T cells versus untransfected controls.

Supplemental PDF S5. Ploidy analysis of *ALPK3*$^{dIC/dIC}$ versus WT.

Supplemental PDF S6. Ploidy analysis of *ALPK3*$^{sIC/sIC}$ versus WT.
